# Bridging Computational Vaccinology and Vaccine Development Through Systematic Identification, Characterization, and Downselection of Conserved and Variable Circumsporozoite Protein CD4 T Cell Epitopes From Diverse *Plasmodium falciparum* Strains

**DOI:** 10.3389/fimmu.2021.689920

**Published:** 2021-06-08

**Authors:** Amy R. Noe, Frances E. Terry, Brian C. Schanen, Emily Sassano, Pooja Hindocha, Timothy W. Phares, Leonard Moise, Jayne M. Christen, Kenneth D. Tucker, Vinayaka Kotraiah, Donald R. Drake, William D. Martin, Anne S. De Groot, Gabriel M. Gutierrez

**Affiliations:** ^1^ Leidos Life Sciences, Leidos Inc., Frederick, MD, United States; ^2^ EpiVax Inc., Providence, RI, United States; ^3^ Sanofi Pasteur, VaxDesign Campus, Orlando, FL, United States; ^4^ University of Georgia Center for Vaccines and Immunology, Athens, GA, United States

**Keywords:** malaria, CSP, *in silico* epitope prediction, HLA-DR, multifunctional cytokine response, cross-strain, TH2R

## Abstract

An effective malaria vaccine must prevent disease in a range of populations living in regions with vastly different transmission rates and protect against genetically-diverse *Plasmodium falciparum* (Pf) strains. The protective efficacy afforded by the currently licensed malaria vaccine, Mosquirix™, promotes strong humoral responses to Pf circumsporozoite protein (CSP) 3D7 but protection is limited in duration and by strain variation. Helper CD4 T cells are central to development of protective immune responses, playing roles in B cell activation and maturation processes, cytokine production, and stimulation of effector T cells. Therefore, we took advantage of recent in silico modeling advances to predict and analyze human leukocyte antigen (HLA)-restricted class II epitopes from PfCSP – across the entire PfCSP 3D7 sequence as well as in 539 PfCSP sequence variants – with the goal of improving PfCSP-based malaria vaccines. Specifically, we developed a systematic workflow to identify peptide sequences capable of binding HLA-DR in a context relevant to achieving broad human population coverage utilizing cognate T cell help and with limited T regulatory cell activation triggers. Through this workflow, we identified seven predicted class II epitope clusters in the N- and C-terminal regions of PfCSP 3D7 and an additional eight clusters through comparative analysis of 539 PfCSP sequence variants. A subset of these predicted class II epitope clusters was synthesized as peptides and assessed for HLA-DR binding *in vitro*. Further, we characterized the functional capacity of these peptides to prime and activate human peripheral blood mononuclear cells (PBMCs), by monitoring cytokine response profiles using MIMIC^®^ technology (Modular IMmune *In vitro* Construct). Utilizing this decision framework, we found sufficient differential cellular activation and cytokine profiles among HLA-DR-matched PBMC donors to downselect class II epitope clusters for inclusion in a vaccine targeting PfCSP. Importantly, the downselected clusters are not highly conserved across PfCSP variants but rather, they overlap a hypervariable region (TH2R) in the C-terminus of the protein. We recommend assessing these class II epitope clusters within the context of a PfCSP vaccine, employing a test system capable of measuring immunogenicity across a broad set of HLA-DR alleles.

## Introduction

The *Plasmodium falciparum* (Pf) circumsporozoite protein (CSP) is a leading target in ongoing malaria vaccine development efforts and the antigenic component of the Mosquirix™ (RTS,S/AS01) vaccine, a recombinant virus-like protein platform containing large portions of the central repeat and C-terminal regions of PfCSP 3D7. Vaccination with RTS,S/AS01 provides moderate protection over a limited amount of time [reviewed in (1)]. In a seven-year assessment of children vaccinated with RTS,S/AS01, efficacies between 31% and 58% were seen in the year post vaccination, with lower efficacy found in vaccinees with high malaria exposure as compared to those with low malaria exposure (2). Additionally, significant reductions in efficacy were found over time, with negative efficacy seen in vaccinees with high malaria exposure between years three and four post vaccination. Efficacy of the RTS,S/AS01 vaccine is also dependent on the infection strain; whereby, efficacy is diminished when vaccinees are infected with a strain of malaria containing a PfCSP allele heterologous to that in the 3D7 vaccine strain (3). Reflective of these findings, there is clear need for PfCSP-based vaccine improvement to achieve sustained and cross-strain protective immune responses.

For PfCSP-based vaccines, both humoral and cellular immune responses to PfCSP are important and have been identified as immunological surrogates of protection in a phase 2 clinical trial where malaria naïve individuals vaccinated with two RTS,S-containing formulations underwent controlled human malaria infection (CHMI) (4). Specifically, geometric mean concentrations of PfCSP-specific antibody were significantly higher in vaccinees protected after CHMI as compared to unprotected vaccinees. Further, significantly greater interferon (IFN)-γ recall responses to a peptide including both hypervariable and conserved residues of PfCSP 3D7 (amino acid address 309-352 per the designations used herein) were seen in protected versus unprotected vaccinees (as assessed *via* ex vivo peripheral blood mononuclear cell (PBMC) ELISpot assay). In this same study, protected vaccinees showed a significantly higher frequency of multifunctional cytokine CD4 T cell responses as compared to unprotected vaccinees (as assessed *via ex vivo* PBMC intracellular staining assay). Of note is that presence of T cell response determinants in the C-terminal region of PfCSP was found in the late 1980s/early 1990s (5–10). Around this time, efforts to sequence and characterize the domains of PfCSP resulted in identification of two highly conserved and two highly variable regions of the protein. The conserved regions, termed Region 1 (R1) and Region 2 (R2), are located slightly N-terminal of the central repeat region (R1) and in the middle of two hypervariable regions in the C-terminal domain of the protein (R2). The two hypervariable regions bracketing R2 were termed TH2R (located N-terminal of R2) and TH3R (located C-terminal of R2). Interest in the C-terminal section of PfCSP containing TH2R, R2, and TH3R intensified when it was determined that these regions contain polymorphic T cell response determinants in malaria-exposed individuals (11). Most relevant to our work is the finding that differential CD4 T cell response profiles were seen with sequence variants of TH2R/R2 region of PfCSP (synthesized as peptides) when assessed ex vivo with PBMCs from malaria-exposed individuals (7–9). While this is the same region of PfCSP to which Kester et al. (4) found differential IFN-γ recall responses in RTS,S vaccinees, significant progress in the comprehensive characterization and comparative analysis of CD4 T cell epitopes within PfCSP has been slow due to the complexities of human leukocyte antigen (HLA)-restriction and lack of tools to predict T cell epitopes.

The advent of computational vaccinology and increasing availability of tools (12, 13) has resulted in an influx of publications utilizing in silico CD4 T cell epitope analysis to rationally design vaccines (14), including those targeting malaria (15–18). Complimentary to this is the curation and compilation of published *in vitro*, ex vivo, and *in vivo* experimental data on T cell epitopes, as part of the Immune Epitope Database and Analysis Resource (IEDB) both for animal models and clinical assessments (19). Further, through recent advances in the overall understanding of humoral immune response development, the central role of follicular helper T cells (Tfh) has emerged with regard to affinity maturation and isotype switching of antigen-specific B cells within germinal centers as well as for the development of protective humoral responses and memory B cells (20). Importantly, recent studies have shown that it is the interplay between Tfh and T follicular regulatory cells (Tfr) that determines the robustness and longevity of the humoral responses (21, 22) and that too strong a T regulatory (Treg) cell response upon vaccination may suppress development of robust long-lived immune responses (23, 24). Additionally, mounting data suggest that induction of Treg responses during malaria infection negatively impacts development of effective immune responses and, along with compromised dendritic cell (DC) function and interference in regulation of immune checkpoint proteins, can lead to chronic disease [reviewed in (24–26)].

It is with this understanding of the gaps in PfCSP-based vaccines, the value of rational vaccine design, and potential immune response inhibition from Treg responses that we undertook an effort to systematically identify and characterize PfCSP CD4 T cell epitopes with the primary goal of identifying class II epitope clusters both present in a large number of PfCSP sequence variants and capable of binding a broad array of HLA-DR alleles. Specifically, we developed an epitope identification and analysis workflow utilizing both the PfCSP 3D7 vaccine strain sequence and 539 publicly-available PfCSP sequence variants (isolated from diverse geographical locations) as sequence inputs. The workflow began with in silico analysis to identify clusters of HLA-DR-restricted epitopes predicted to bind promiscuously across a broad panel of HLA-DR alleles. This was followed by laboratory assessments to validate our HLA binding predictions against *in vitro* HLA-DR allele binding and the subsequent use of these results to curate class II clusters of interest. Lastly, we assessed the ability of the predicted epitope clusters, synthesized as peptides, to prime human DCs and T cells as well as to elicit multifunctional cytokine responses.

## Materials and Methods

### PfCSP Custom Sequence Database

Publicly-available PfCSP sequences were collected from UniProt on June 22, 2016, and compiled in a database including metadata associated with geographic origin and genotypic space. A total of 540 sequences were retrieved; however, one sequence was excluded due to the lack of a geographical tag. The final database comprised 539 amino acid sequences of variable lengths (PfCSP sequence variants). Due to incomplete representation of portions of the CSP protein preceding the repeat domain (**Figure S1**), the database was subdivided into two reference sets reflecting the most complete set of N-terminal and C-terminal sequences, respectively. The N-terminal reference set contained 329 sequences, while the C-terminal reference set contained 525 sequences. Information regarding the geographic origin and distribution of the 539 PfCSP sequence variants is shown in **Figure S2**.

### *In Silico* T Cell Epitope Prediction and Analyses

The in silico analysis was conducted by EpiVax utilizing several tools from their iVAX toolkit (12, 27). Using the EpiMatrix tool, input amino acid sequences were parsed into overlapping 9-mer frames and each frame evaluated for predicted binding to a panel of nine class II HLA-DRB1 alleles (*0101, *0301, *0401, *0701, *0801, *0901, *1101, *1301, and *1501). EpiVax utilizes these alleles for their binding prediction algorithms as they represent functional allele supertypes (i.e., HLA alleles clustered into families based on the ability to bind peptides with related amino acid sequences) capable of evaluating predictive immunity to over 95% of the global human population regarding HLA supertypes (28, 29). EpiVax normalizes these HLA binding predictions as EpiMatrix Z-scores (the output of this tool) to enable comparisons across alleles, and identifies significant frame “hits” by applying a Z-score cutoff of 1.64 (the top 5% of binding frames from a dataset of 10,000 random sequences) to signify a high probability of HLA allele binding. EpiVax also designates Z-scores in the top 1% of binding frames (Z-scores >2.32) as hits with the highest probability of binding. The ClustiMer tool utilizes the EpiMatrix output to identify regions of high epitope density in the input sequences and defines class II HLA epitope clusters, which consist of a binding core (containing a high density of predicted epitopes across the set of HLA-DR alleles evaluated) and flanking amino acids (30). In addition, the resulting predicted epitope sequences were evaluated for homology to the human genome (i.e., extent of “human-ness”) as an indicator of the potential to generate immunosuppressive responses including autoimmune or Treg responses. This homology analysis is performed using the JanusMatrix tool, which examines human sequence similarity with respect to the HLA and T cell receptor (TCR) faces of an epitope to flag sequences that could potentially elicit undesired immunosuppressive responses due to homology with sequences encoded by the human genome (31). The JanusMatrix (human homology) score of a given amino acid sequence indicates the number of potential immunosuppressive response triggers or flags, with higher JanusMatrix scores indicating a bias towards immune tolerance (32). Ninety-five percent of randomly generated predicted ligands to HLA-DRB1 supertype alleles have JanusMatrix (human homology) scores between zero and two. Therefore, JanusMatrix scores greater than two are the established threshold for flagging the extent of human-ness in a sequence. Note that additional information concerning the role of the Treg repertoire in maintenance of self-tolerance can be found in Feng et al. (33). Additional tools utilized for this analysis include those to interrogate PfCSP sequence variants within the custom sequence database developed for this project. These tools included Conservatrix, which identifies predicted epitopes that are conserved across a set of sequence variants, and the EpiAssembler tool, which works to identify class II HLA epitope clusters across a set of sequence variants by assembling overlapping predicted epitopes into immunogenic consensus sequences (ICS) (34). EpiVax provided detailed EpiMatrix outputs listing Z-scores for each frame across the complete set of HLA-DR alleles evaluated. We summarized these data as the highest Z-score and total number of predicted epitopes (EpiMatrix hit count) by HLA allele and PfCSP class II cluster **(** **Table S1** **).**

### Peptide Synthesis

Predicted class II HLA epitope clusters were synthesized as peptides using solid phase chemistry, 9-fluoronylmethoxycarbonyl synthesis, by 21st Century Biochemicals (Marlborough, MA). Peptides were delivered >85% pure for *in vitro* assays and >95% pure for *ex vivo* assays, as ascertained by HPLC, mass spectrometry and UV scan to verify purity, mass, and spectrum, respectively. In all cases, the amino acid content of each peptide was determined to enable reconstitution at highly accurate molarity; therefore, in some cases, peptides were synthesized without an N-terminal acetyl group (all peptides were synthesized with C-terminal amino group caps), with the addition of flanking lysine residues to add charge, and/or trimmed from the ClustiMer output to facilitate peptide synthesis/purification and/or adhere to the established solubility parameters. Two peptides were synthesized without an N-terminal acetyl group to facilitate synthesis and purification. In order to establish a net charge for two of the peptides, lysine flanking residues were added. In addition, for three peptides, the sequence was trimmed from the ClustiMer output as shown in **Table S2** (further see **Table S5** for peptide sequences).

### *In Vitro* HLA Binding Assays

EpiVax conducted *in vitro* quantification of peptide-HLA binding affinity utilizing a competition assay format per the methodology described in (35). Briefly, a fluorescent-labeled, high-binding reference peptide and titrating concentrations of test peptide were incubated in a 96-well plate format with limiting concentrations of class II HLA monomers in aqueous buffer for 24-hours. Post incubation, the mixtures were moved to a 96-well plate coated with anti-HLA-DR antibody to capture HLA-peptide complexes. Time-resolved fluorescence measurement of the bound labeled reference peptide complex present in each mixture were detected with a europium-linked probe *via* fluorescent spectrophotometry using a SpectraMax M5 system. HLA binding affinity of each test peptide was expressed as the percent inhibition of reference peptide binding. Percent inhibition values (across the test peptide titration range) were used to calculate the half maximal inhibitory concentration (IC_50_) of each test peptide. For these studies, test peptides were assessed using a range of final concentrations from 100,000 nM to 100 nM and all peptides were reconstituted in dimethyl sulfoxide (DMSO). The panel of commercially-available HLA-DRB1 allele monomers used included: *0101, *0301, *0401, *0701, *0801, *1101, *1301 and *1501.

### *Ex Vivo* CD4 T Cell Simulation Assays Using the MIMIC^®^ Platform

For MIMIC (Modular IMmune *In vitro* Construct) platform studies, PBMCs from healthy donors enrolled in a Sanofi Pasteur-VaxDesign campus apheresis program were used. All blood samples obtained and used for this effort were collected from consenting participants in compliance with an institutional review board (IRB)-approved protocol (CRRI 0906009). Within hours following harvest from the donor, the enriched leukocytes were centrifuged over a ficoll-plaque PLUS (GE Healthcare, Piscataway, NJ) density gradient. PBMCs at the interface were collected, washed, cryopreserved in IMDM media (Lonza, Walkersville, MD) containing autologous serum and DMSO (Sigma–Aldrich, St. Louis, MO) and stored in vapor phase liquid nitrogen until needed.

Monocytes were purified from total PBMCs by anti-CD14 antibody-conjugated magnetic beads (Stemcell Technologies, Cambridge, MA), and cultured at 1 million cells per mL for 6 days in serum-free CellGro DC Medium (CellGenix, Portsmouth, NH) supplemented with 100 ng/mL GM-CSF (R&D Systems, Minneapolis, MN) and 25 ng/mL interleukin (IL)-4 (R&D Systems). DCs were matured using 10ng/mL of LPS (Sigma-Aldrich) and 100IU/mL of IFN-γ (PeproTech, Rocky Hill, NJ). The matured DCs were then harvested for assay use in the CD4 T cell stimulation assay within 16 hours of maturation.

The CD4 T cell stimulation assays were performed using protocols established at Sanofi Pasteur–VaxDesign Campus (36, 37). Autologous CD4 T cells were enriched from frozen PBMCs by negative magnetic bead selection (Stemcell Technologies) and then co-cultured at 2 million T cells per well with autologous DCs at a ratio of 60:1 in X-VIVO 15 media (Lonza). Prior to use, DCs were pre-pulsed for at least 2 hours with pooled peptides (two peptides per pool used at a concentration of 5 µg/mL for each peptide).

After a 14-day incubation period, lymphocytes were harvested and evaluated for effector activity using intracellular cytokine staining (ICCS). For the ICCS assay, autologous pre-pulsed DCs were co-cultured with the harvested primed T cells for 7 hours. 1µg/mL brefeldin A (Sigma–Aldrich) was added for the final 5 hours of culture to prevent protein egress from the Golgi apparatus. Following the incubation period, cells were labeled with the Live/Dead Fixable Stain Kit (Invitrogen, Carlsbad, CA), treated with cytofix/cytoperm and permwash reagents from BD Biosciences (San Jose, CA), and then labeled with Bioscience (San Diego, CA) antibodies specific for human IFN-γ, tumor necrosis factor (TNF)-α, IL-2, IL-4, IL-10, and CD154. The samples were then acquired on an LSRII flow cytometer (BD Biosciences) and analyzed using FlowJo software (TreeStar, Ashland, OR). The CEF-MHC class II control peptide pool “plus” (Cellular Technology, Ltd.) was used as a positive control for the assay at the manufactures suggested concentration of 8 μg/mL. This positive control peptide pool contains 23 known MHC class II epitopes derived from human Cytomegalovirus, Epstein Barr virus, influenza virus, and tetanus toxin. Stimulation Index was calculated as the response obtained in pre-pulsed cells stimulated with peptide divided by the response seen in pre-pulsed cells with no peptide added during the stimulation phase.

## Results

### Identifying Input Sequences for Analysis and Developing the Experimental Workflow

**Figure 1:**
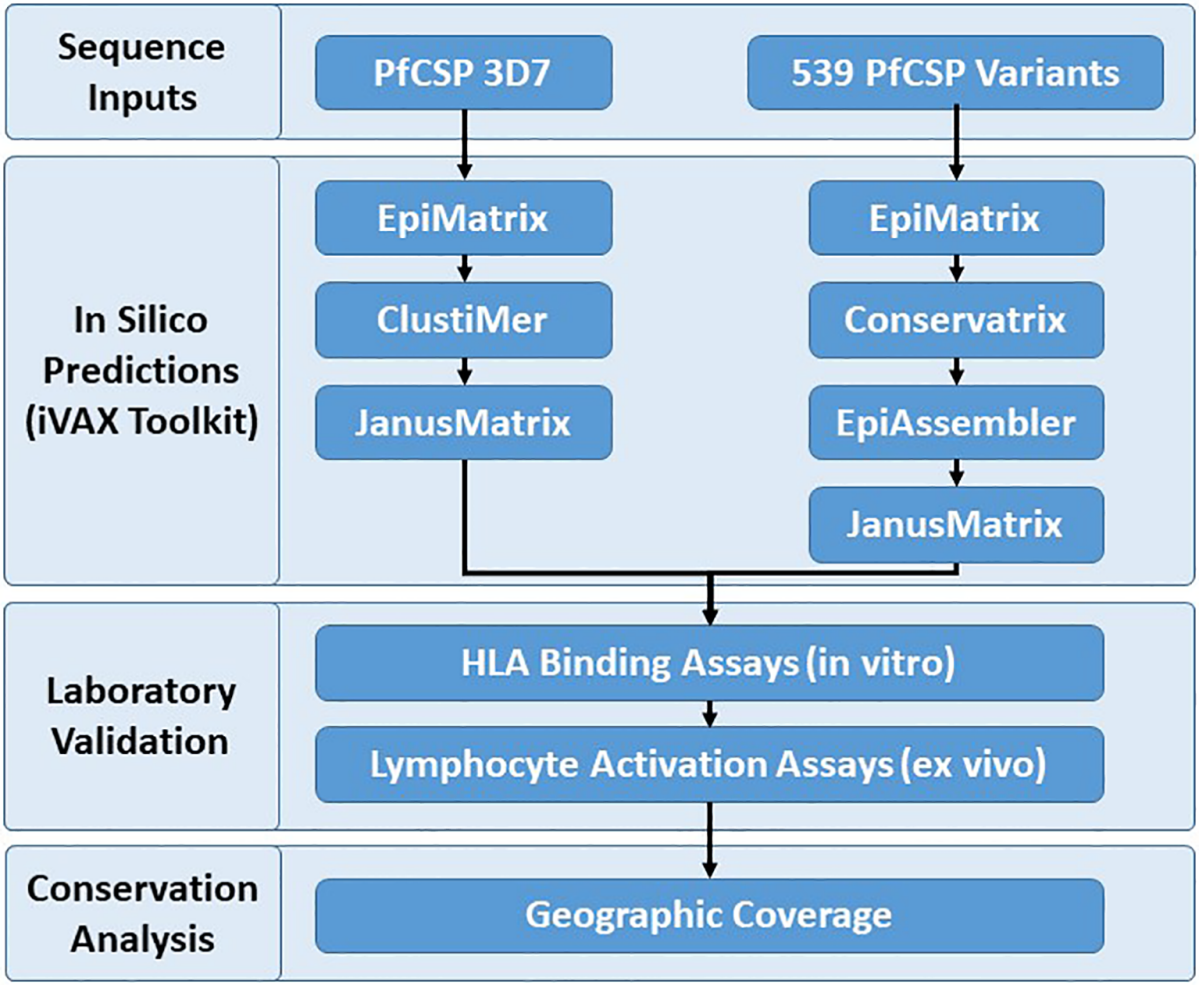
Experimental Workflow.

Identification and assessment of the human T cell epitopes within PfCSP 3D7 and PfCSP variants were performed to better understand vaccine candidate sequences that impact cell-mediated immunity across different HLA alleles with the goal of improving PfCSP-based malaria vaccines. The PfCSP 3D7 sequence was selected for single protein analysis because this is the vaccine strain. A reference database of 539 PfCSP sequence variants was developed in order to assess coverage of putative class II T cell epitopes in PfCSP 3D7 across the geographic and genotypic space represented by the custom sequence database, as well as to identify additional putative epitopes among PfCSP variants. The PfCSP 3D7 sequence (PF3D7_0304600) and the 539 PfCSP sequence variants served as input sequences for the experimental workflow (**Figure 1**) that started with in silico analyses, moved to *in vitro* and *ex vivo* laboratory assessments, and then to an epitope conservation analysis to facilitate final downselection. Specifically, the experimental workflow was as follows (1): in silico analysis of the PfCSP 3D7 protein sequence using EpiMatrix to identify predicted class II T cell epitopes, ClustiMer to define class II HLA clusters by identifying regions of high epitope density, and JanusMatrix to assess extent of human-ness as a flag for potential immunosuppressive responses; (2) in silico analysis of the 539 variant PfCSP protein sequences using EpiMatrix to identify predicted class II T cell epitopes, Conservatrix to identify conservation across the predicted epitopes, EpiAssembler to identify class II HLA-DR epitope clusters across the set putative epitopes by assembling overlapping predicted epitopes into ICS, and JanusMatrix to assess the extent of human-ness; (3) *in vitro* HLA-DR binding assays of the putative class II epitope clusters (as peptides); (4) *ex vivo* multifunctional cytokine T cell simulation of human PBMCs with the putative class II epitope clusters (as peptides); and (5) epitope conservation analysis to evaluate the PfCSP sequence variants, in consideration of epitope coverage and geographical region, to aid in final downselection.

### *In Silico* T Cell Epitope Analysis of the PfCSP 3D7 Protein Sequence

**Figure 2:**
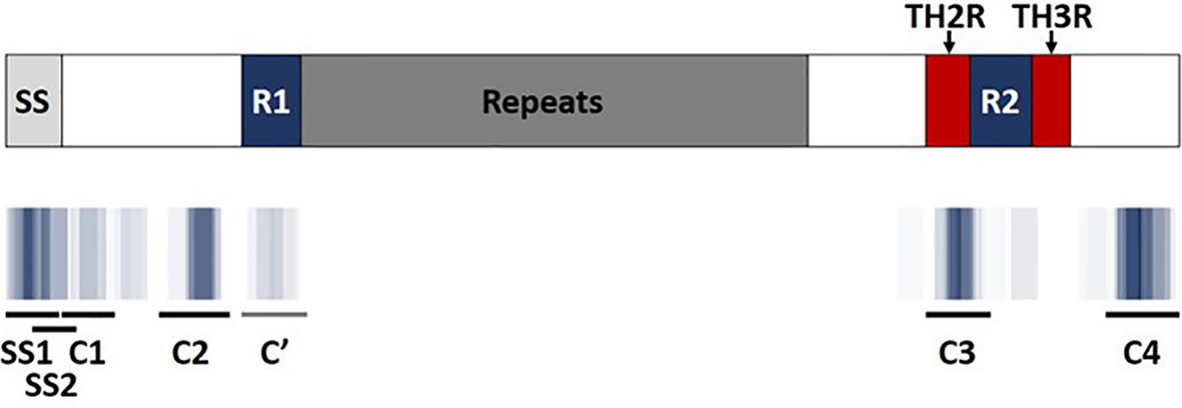
EpiMatrix predicted class II T cell epitope coverage for PfCSP 3D7. Major regions of PfCSP (top) are shown aligned with predicted class II T cell epitope clusters (bottom). Areas of darker blue indicate higher numbers of predicted epitopes while white areas indicate a lack of predicted epitopes. ClustiMer outputs are shown as black and grey lines where C1-C4 represent the four identified epitope clusters, non-inclusive of the two predicted clusters overlapping the signal sequence (SS). C’ represents a pseudo-cluster containing only a few predicted epitopes. The repeat region, highly conserved regions (R1 and R2), and highly variable regions (TH2R and TH3R) of CSP are depicted.

Promiscuous class II T cell epitopes predicted to bind across a broad set of HLA-DR alleles (DR1, DR3, DR4, DR8, DR9, DR11, DR13, and DR15) were identified using the EpiMatrix and ClustiMer algorithms. These specific alleles were selected because they are the most common HLA alleles within each of the HLA supertypes (38, 39) and are representative of >95% of human populations worldwide without the need to test each individual haplotype (28, 29). EpiMatrix algorithm output provided Z-scores indicating the predicted binding of each overlapping 9-mer in the PF3D7_0304600 amino sequence to each of the HLA alleles in the panel. The Z-scores equal to or greater than 1.64 represent the top 5% of predicted binding frames and signify a 9-mer with a high probability HLA allele binding. A composite representation of the predicted class II epitopes in PfCSP 3D7 (i.e., 9-mers with EpiMatrix Z-scores ≥ 1.64) across all nine class II HLA alleles is shown in **Figure 2**. Although class II HLA-DR molecules bind a 9-mer sequence (i.e., the binding core), clustering of binding cores within antigens is typical and sequences flanking the binding core can impact HLA binding (40). Within PfCSP 3D7, the ClustiMer algorithm identified a total of six class II epitope clusters, two in the signal sequence (SS1 and SS2), two in the N-terminal region of CSP (C1 and C2), and two in the C-terminal region of CSP (C3 and C4). In addition, a pseudo-cluster overlapping the R1 domain of CSP was also defined and designated as C’. Except C1, which did not contain any predicted epitopes for two of the HLA-DRB1 alleles, and the pseudo-cluster (C’), which did not contain any predicted epitopes for four of the HLA-DRB1 alleles, all of the designated clusters contained at least one predicted epitope for each of the nine HLA-DRB1 alleles (**Table S1**). The sequences for all six clusters and the pseudo-cluster along with their respective EpiMatrix (EPX) and JanusMatrix (JMX) cluster scores are shown in **Table 1**. The EpiMatrix cluster score represents predicted immunogenic potential of an epitope cluster sequence, based on the number of predicted epitopes within the sequence and their relative Z-scores. EpiMatrix cluster scores above ten are comparable to those of known promiscuous HLA-DRB1 epitopes (**Figure S3**) and all six PfCSP 3D7 clusters, but not the pseudo-cluster, reached this threshold (**Table 1**). The JanusMatrix cluster score represents the extent of human-ness in the epitope cluster sequence (i.e., predicted synonymous HLA-DRB1 binding frames with TCR-binding face residues that match the human proteome). JanusMatrix cluster scores greater than 2.0 suggest increased potential for an immunosuppressive response (e.g., a Treg response). Two of the PfCSP 3D7 clusters (SS1 and C2) and the pseudo-cluster (C’) exceeded this threshold (**Table 1**). Importantly, an over threshold JanusMatrix cluster score is intended to make the researcher aware of the human proteome overlap; however, the actual elicitation of Treg responses must be determined empirically. Additional information regarding the JanusMatrix output is provided in **Table S2**. With regard to clusters SS1 and SS2, as CSP is already on the surface of sporozoites within the mosquito salivary glands, the predicted clusters overlapping the signal sequence were not selected for further analysis. In addition, given that the goal of the in silico analysis was identification of promiscuous class II T cell epitopes, the pseudo-cluster was not selected for further analysis as it contained a limited number of predicted epitopes. Thus, based on these in silico assessments, clusters C1-C4 were selected for further analysis.

**Table 1:** Clusters of Predicted Class II Epitopes within PfCSP Associated with EpiMatrix (EPX) and JanusMatrix (JMX) Cluster Scores.

Cluster ID	Address/Strain^a^	Cluster Sequence^b^	EPX Cluster Score	JMX Cluster Score	Conserved (Y/N)
SS1	1 – 18 (3D7)	**MMRKLAILSVSSFLF**VEA	40.70	3.57	Y
SS2	10 – 24 (3D7)	VSS**FLFVEALFQ**EYQ	19.64	0.90	Y
C1	20 – 37 (3D7)	FQE**YQCYGSSSNTRV**LNE	12.03	0.13	Y
C2	53 – 76 (3D7)	MNY**YGKQENWYSLKKNSRSLG**END	40.05	2.59	Y
C’	80 – 103 (3D7)	NED**NEKLRKPKHKKLKQPADG**NPD	6.26	3.88	Y
C3	313 – 334 (3D7)	DKH**IKEYLNKIQNSLSTEW**SPC	41.17	2.00	N
C4	374 – 397 (3D7)	CSS**VFNVVNSSIGLIMVLSFLFLN**	65.95	1.22	Y
ICS1^c^	374 – 397 (3D7)	CSS**VFNVVNSSIGLIMVLSFLFLN**	65.95	1.22	Y
ICS2	TH2R/R2 Variant	ITD**YLKKIQNSLSTEWS**PCS	45.19	4.00	N
ICS3	TH2R/R2 Variant	DQH**IEQYLKKIQNSIST**EWS	35.01	2.24	N
ICS4	TH2R/R2 Variant	DQH**IEQYLKTIQNSLST**EWS	27.15	2.39	N
ICS5	TH2R/R2 Variant	DQH**IEKYLKIIQNSLST**EWSP	47.79	1.89	N
ICS6	TH2R/R2 Variant	IKK**YLKKIKNSISTEWS**PCS	41.12	2.93	N
ICS7	TH2R/R2 Variant	IEQ**YLKKIQYSLSTEWS**PC	27.04	2.10	N
ICS8^d^	313 – 334 (3D7)	DKH**IKEYLNKIQNSLSTEW**SPC	41.17	2.00	N
ICS9	TH2R/R2 Variant	DKH**IEKYLKRIQNSLST**EWS	41.67	2.79	N
ICS10	TH2R/R2 Variant	DQH**IEKYLKTIKNSLST**EWS	36.14	2.35	N

a Amino acid address for the 3D7 strain or TH2R/R2 variant is indicated.

b Amino acid sequences for the HLA binding cores (bold) and flanks are shown.

c ICS1 sequence output from the EpiAssembler analysis matched the C4 sequence (3D7).

d ICS8 sequence output from the EpiAssembler analysis matched the C3 sequence (3D7).

### *In Silico* T Cell Epitope Analysis of PfCSP Sequence Variants

In addition to the class II clusters within PfCSP 3D7, identification of additional predicted T cell epitopes within the reference database sequences (539 PfCSP variants) was performed utilizing the EpiMatrix, JanusMatrix, Conservatrix, and EpiAssembler algorithms. For this analysis, the level of PfCSP variant sequence cross-conservation was assessed with these tools to identify predicted epitopes that are conserved across the set of sequence variants. Output from this analysis, when comparing the database of PfCSP sequence variants to the PfCSP 3D7 sequence, showed three clusters of predicted class II epitopes with particularly well conserved TCR contours, one cluster with moderately conserved TCR contours, and one cluster with poorly conserved TCR contours (**Table S3**). The three highly-conserved clusters (which overlap the positions 20 – 37, 53 – 76, and 80 – 103 of PfCSP 3D7, or C1, C2, and C’, respectively) are identical at all TCR-facing positions and conserved at the HLA binding positions with respect to >80% of the PfCSP sequence variants. Thus, we anticipate that T cell responses targeting these sequences may also target homologous sequences from many PfCSP variants. The one moderately conserved cluster (which overlaps the position 374 – 397 of PfCSP 3D7 or C4) contains TCR- and HLA-facing residues conserved with respect to 62% of the PfCSP sequence variants. Lastly, the cluster with poorly conserved TCR contours overlaps the position 313-334 of PfCSP 3D7 (or C3) and is quite variable, as these residues intersect the CSP TH2R domain. The TCR- and HLA-facing residues of the PfCSP 3D7 C3 sequence are only conserved with respect to 19% of PfCSP sequence variants. Therefore, we anticipate T cell responses targeting this sequence may stimulate T cells that only react to a limited number of other PfCSP variants. Given the lower cross-conservation in the C-terminal region of the PfCSP sequence variants, we anticipated that additional predicted class II epitopes might be found in this region of the protein. Therefore, Conservatrix outputs were applied for the EpiAssembler analysis to identify class II HLA epitope clusters across the set of PfCSP sequence variants by assembling overlapping predicted epitopes into ICS representing the class II epitope clusters with the highest predicted immune potential from 539 PfCSP sequence variants (with 329 variants including residues in the N-terminal region of the protein and 525 variants include residues in the C-terminal region of the protein). Output from the EpiAssembler analysis yielded a total of three ICS from the N-terminal region of PfCSP – these were identical to the predicted PfCSP 3D7 clusters (i.e., C1, C2, and ‘C) – and ten ICS from the C-terminal region of PfCSP, two of which were identical to the predicted PfCSP 3D7 clusters (i.e., ICS1 = C4 and ICS8 = C3) and eight of which were novel clusters that contained variants of the TH2R domain sequence (**Table 1**). Regarding the eight novel clusters, all eight had EpiMatrix cluster scores above ten, suggesting good immune potential, and seven of these had JanusMatrix cluster scores above the 2.0 threshold (i.e., human-ness flags suggesting increased potential for a Treg response). Additional information regarding the JanusMatrix output is provided in **Table S2**.

### *In Vitro* HLA-Peptide Binding Analysis

**Figure 3:**
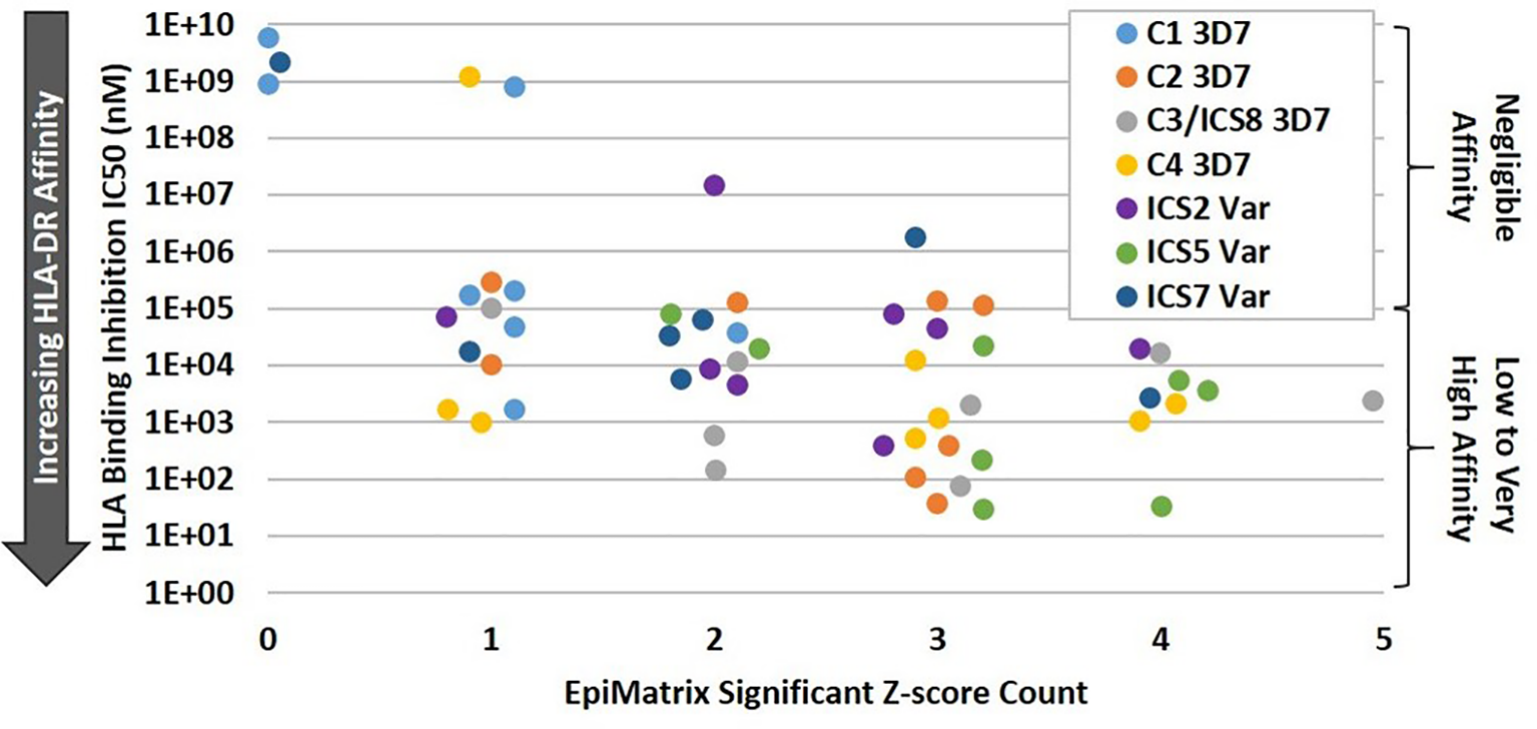
Increasing HLA-DR Allele Binding Affinity Trends with Increasing EpiMatrix Significant Z Score Count. For the class II clusters C1-C4, ICS2, ICS5, and ICS7, *in vitro* HLA binding affinity to DR1, DR3, DR4, DR8, DR11, DR13, and DR15 alleles was plotted against the number of in silico predicted epitopes for the subject HLA-DR. In general, higher *in vitro* HLA-DR affinity was seen for those alleles with higher numbers of in silico predicted epitopes within a cluster.

In order to validate the in silico HLA binding predictions, a subset of the predicted class II T cell epitope clusters were synthesized as peptides and evaluated for HLA-DRB1 allele binding using an *in vitro* competition assay whereby test peptides compete for binding with a fluorescently-labeled positive control peptide known to strongly bind the subject HLA-DRB1 allele (**Table S4**). The subset of clusters selected for this analysis included the four PfCSP 3D7 clusters of interest (C1-C4) and three ICS (ICS2, ICS5, and ICS7). As ICS2-ICS10 (the TH2R/R2 variants) have some sequence overlap, the three ICS were selected based on variation in the HLA-facing residues as compared to C3. The seven clusters (synthesized as peptides) were assessed for binding using a panel of eight HLA-DRB1 alleles that included: *0101, *0301, *0401, *0701, *0801, *1101, *1301 and *1501. Note that HLA-DRB1*0901 was not included in the panel due to the lack of a suitable commercially-available reagent. For each test peptide, the concentration that inhibited 50% of the specific HLA binding by the control peptide was calculated as IC_50_. These IC_50_ values were used to gauge affinity of the test peptide, with lower IC_50_ values indicating greater affinity (**Table S5**). To better understand the level of concordance between *in vitro* HLA binding and in silico HLA binding prediction, IC_50_ was plotted against the significant EpiMatrix Z score count (i.e. number of predicted epitopes) for each cluster by HLA-DR. We found that increased binding affinity for an HLA-DR allele trended with increased numbers of significant Z score counts for the associated HLA-DR allele (**Figure 3**). Overall, an accuracy of 79% was found between the in silico HLA-DR binding predictions and the *in vitro* HLA-DR allele binding for the peptides tested (**Table S6**). Given the relatively low significant Z score counts for C1 (**Table S1**) and overall lack of high affinity HLA binding with this sequence (**Table S5**), the C1 cluster was dropped from further study.

### *Ex Vivo* Human PBMC Immunogenicity Assessments

**Figure 4:**
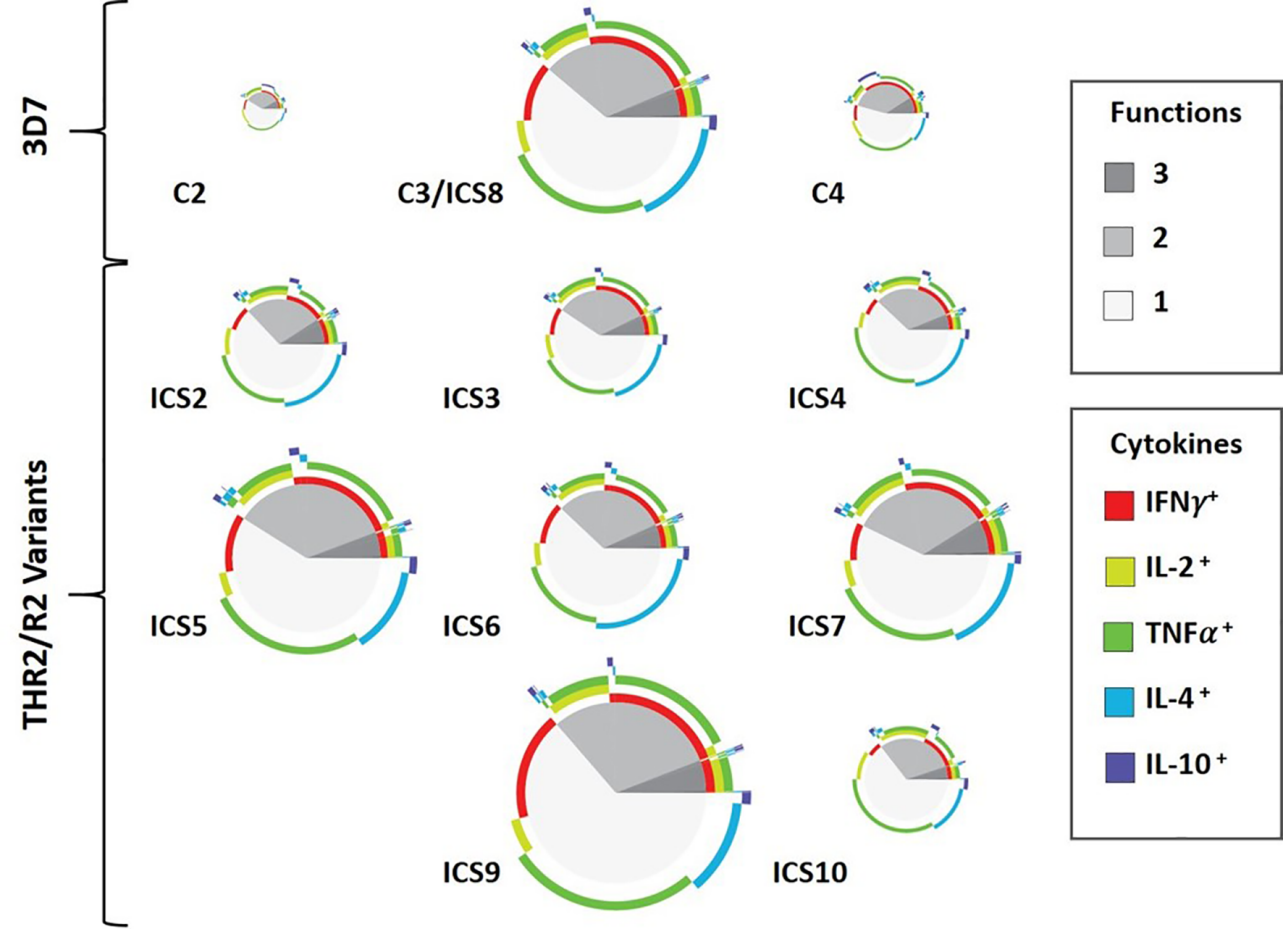
Multifunctional Cytokine-Producing CD4 T Cell Response Profiles to Peptides Comprised of PfCSP-Predicted Epitope Clusters. Circular pie charts represent median response across all 30 donors (i.e., individual pie charts represent the median value of the summation of total magnitude for the combined cytokine responses from all the donors for each peptide). The proportion of single-, double-, and triple-function T cells is shown in grey tones (pie slices). The colored arcs highlight the total proportions of T cells secreting a particular cytokine or combination of cytokines. Relative magnitude of the overall median response (for all 30 donors to a peptide) can be gauged by size of the pie chart, with greater magnitude response represented by larger pie charts.

The eleven class II epitope clusters of interest (C2, C3/ICS8, C4/ICS1, ICS2-ICS7, ICS9, and ICS10) were assessed for the ability to elicit recall responses using the MIMIC^®^ platform. This platform simulates immune responses from a diverse human population using the PBMCs of individual donors to recapitulate each individual’s human immune response (37). Autonomy of each donor is maintained resulting in an *ex vivo* test system that is functionally equivalent to the donor’s own immune system and designed to respond in a similar manner as that seen *in vivo*. One key aspect of this platform is that the lymphoid tissue equivalent (LTE) module simulates adaptive immune responses through DC priming and production of activated T cells and cytokines, to mirror cellular immune response within a human lymph node. In other words, the cells of PfCSP naïve donors can be used in this platform, as the LTE module provides the mechanism for DC antigen presentation and T cell priming to the peptides of interest. Further, assessment of recall multifunctional cytokine responses (to the peptides) is then possible utilizing the primed DC/T cell co-cultures. For each peptide, the number and magnitude of CD4 cytokine-producing T cells was evaluated across a panel of five cytokines, IFN-γ, TNF-α, IL-2, IL-4, and IL-10. This panel included T helper cell type 1 (Th1) response markers (IFN-γ, TNF-α, and IL-2), a T helper cell type 2 (Th2) response marker (IL-4), and a marker for Treg response (IL-10). Overall, the most prevalent Th1 responses were IFN-γ, followed by TNF-α and then IL-2. The percentage of donors with IFN-γ recall responses to each peptide is shown for each HLA allele in **Table 2**. Positive responses were calculated based on a stimulation index (SI) of 1.5-fold above the baseline. For IFN-γ recall responses the median SI was the lowest for ICS4 (0.74) and the highest for ICS7 (1.75), while the SI range across all donor/peptide combinations was 0.17 to 24.05. For reference, a representative set of flow cytometry CD4+CD154+IFN-γ+ cytokine responses is provided in **Figure S4**. With one exception (ICS4), the highest percentage of donors with IFN-γ responses across the HLA-DR allele set were seen with the TH2R/R2 variants (i.e., C3/ICS8, ICS2, ICS3, ICS5-ICS7, ICS9, and ICS10). Similar results were found for TNF-α and IL-2 (**Tables S7** and **S8**, respectively). With regard to Th2 responses, a relatively low percentage of IL-4 recall responses were seen (**Table S9**). This was also the case for the Treg response marker, in that a relatively low percentage of donors showed IL-10 recall responses (**Table S10**). Of note is that C2 and ICS6 had the highest percentage of IL-10 recall responses across the donor set. Further, the JanusMatrix cluster scores for these two sequences were elevated, 2.59 and 2.93, respectively. While ICS2 and ICS10 also had elevated JanusMatrix cluster scores, the percentage of IL-10 recall responses seen to these peptides was not as high. In addition to evaluating donor responses to individual cytokines, we also looked at multifunctional T helper cell cytokine profiles, which serve as the primary readout of T cell immunogenicity assessments performed with the MIMIC^®^ LTE module. Multifunctional response profiling of the generated T cell sets was performed by multilayered Boolean data analysis; whereby, data are represented as circular pie charts showing the number of functions (i.e., the number of cytokines secreted) in grey, with darker colors denoting an increased number of functions, and type of function (i.e., cytokine secreted) in color, shown as broken concentric circles (**Figure 4**). A total of 31 individual cytokine combinations of IFN-γ, TNF-α, IL-2, IL-4, and IL-10 produced by individual CD4 T cells were possible. The proportion of the total response of each cytokine alone or in any combination produced at the single-cell level reflects the quality of the response. Additionally, the overall magnitude of the combined cytokine response is represented for each peptide by size of the circular chart. Based on the percentage of donor recall responses, multifunctional response profiles, and magnitude of the overall cytokine responses, the standouts from the MIMIC^®^ platform assessment were C3/ICS8, ICS5, ICS7, and ICS9.

**Table 2:** Percentage of Donors by HLA Type Demonstrating IFN-γ Responses to Peptides Comprised of PfCSP Predicted Epitope Clusters.

Cluster ID	HLA Allele								
	DR1	DR3	DR4	DR7	DR8	DR9	DR11	DR13	DR15
**C2**	**20%**	**33%**	**0%**	**0%**	**0%**	**25%**	**0%**	**0%**	**29%**
**C3/ICS8**	**20%**	**33%**	**50%**	**50%**	**75%**	**50%**	**25%**	**0%**	**29%**
**C4**	**20%**	**17%**	**30%**	**0%**	**25%**	**0%**	**50%**	**29%**	**0%**
**ICS2**	**40%**	**33%**	**40%**	**50%**	**50%**	**0%**	**25%**	**57%**	**43%**
**ICS3**	**80%**	**33%**	**30%**	**75%**	**0%**	**0%**	**25%**	**0%**	**14%**
**ICS4**	**0%**	**0%**	**30%**	**0%**	**0%**	**0%**	**0%**	**0%**	**29%**
**ICS5**	**80%**	**33%**	**60%**	**75%**	**25%**	**50%**	**100%**	**43%**	**43%**
**ICS6**	**20%**	**50%**	**40%**	**50%**	**25%**	**50%**	**50%**	**43%**	**43%**
**ICS7**	**60%**	**67%**	**70%**	**75%**	**75%**	**50%**	**25%**	**86%**	**43%**
**ICS9**	**40%**	**50%**	**70%**	**75%**	**50%**	**50%**	**75%**	**43%**	**57%**
**ICS10**	**20%**	**33%**	**40%**	**75%**	**0%**	**25%**	**50%**	**29%**	**57%**
**N**	**5**	**6**	**10**	**4**	**4**	**4**	**4**	**7**	**7**

A Stimulation Index greater or equal to 1.5-fold above baseline is used to specify a positive donor response. The percentage of responding donors, by HLA allele for each predicted 3D7 and ICS cluster, is shown numerically along with color-coding to indicate higher percentages of responders with darker blues. The number of HLA-matched donors (N) for each allele is shown in the bottom row. A total of 30 donors were included in this study.

### Sequence Conservation and Predicted Coverage for TH2R/R2 Sequence Variants

**Figure 5:**
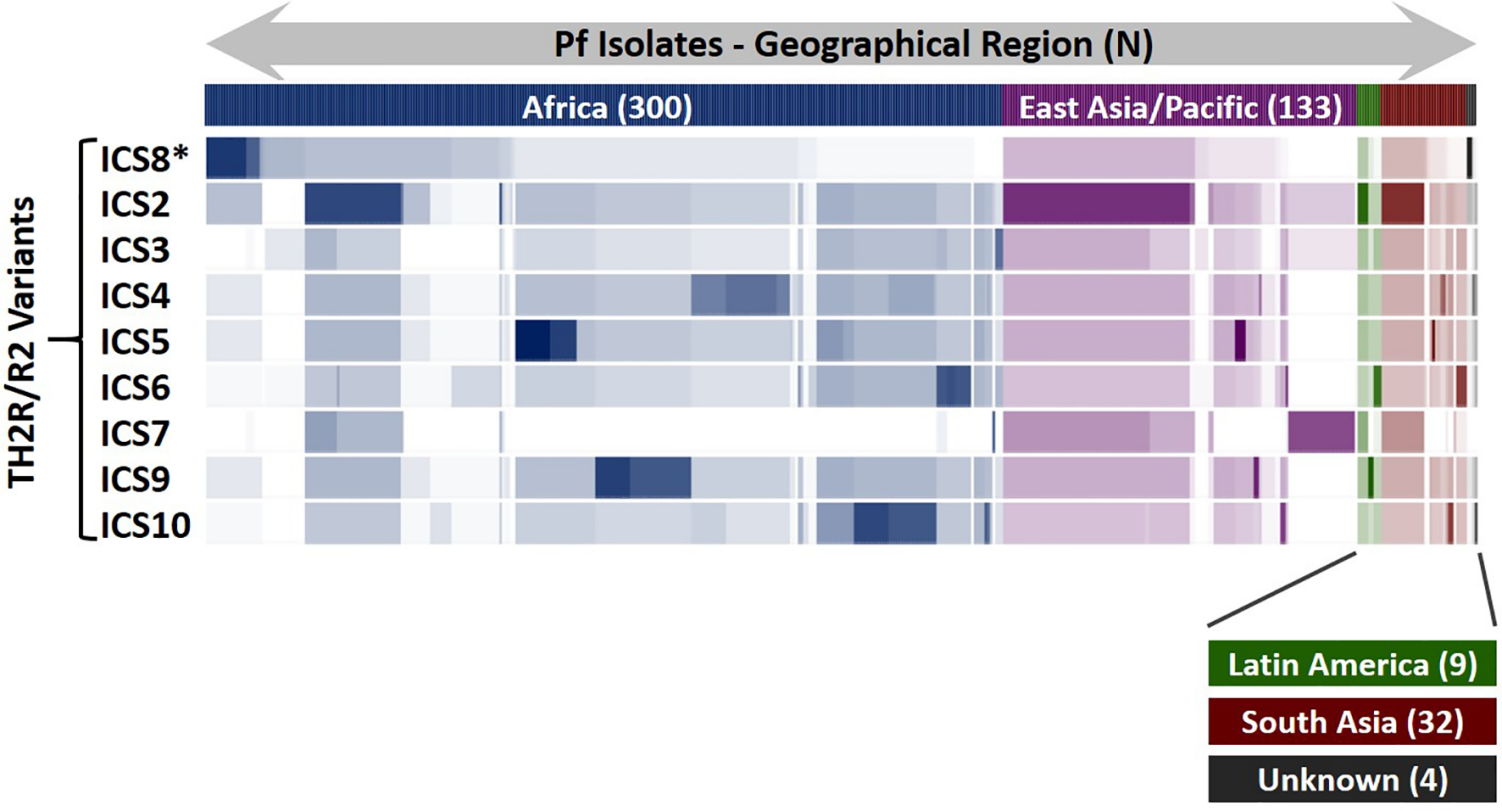
PfCSP Isolate Coverage Map for TH2R/R2 Epitope Cross-Conservation. An epitope sequence cross-conservation analysis was performed to determine the number of matching predicted epitopes between each ICS and the 478 PfCSP isolates evaluated to identify the ICS. The Pf isolates are shown horizontally, grouped by geographical region, and the number of isolates for each geographical region (N) is indicated. Areas of darker colors indicate higher numbers of matching predicted epitopes while white areas indicate a lack of matching predicted epitopes. *ICS8 is the 3D7 sequence and identical to the C3 sequence.

To assist with final class II cluster downselection, an epitope sequence conservation analysis was performed to evaluate epitope coverage and geographic region for sequences within the custom database relative to the TH2R/R2 variants. In total, 478 PfCSP sequence variants contained the full PfCSP TH2R/R2 domains and were used for this analysis, matching the predicted epitopes within each of the ICS to the set of 478 variants. Results of this analysis are represented as a heat map showing the number of epitope matches for each PfCSP sequence variant along with the geographic region where the sequence variant was isolated (**Figure 5**). Of note is that ICS2 has epitope conservation across a broad geographic range, including PfCSP variants isolated in Africa, East Asia/Pacific, Latin America, and South Asia. Further, ICS7 has epitope conservation with a set of East Asia/Pacific origin variants not seen in the other ICS. In consideration of T cell recall response cytokine profiles and epitope conservation across PfCSP sequence variants, respecting both geographical origin and number of variants, we recommend the following class II epitope clusters for future PfCSP vaccine development: C3/ICS8, ICS2, ICS5, ICS7, and ICS9.

## Discussion

We systematically identified and characterized a set of PfCSP class II epitope clusters by establishing a workflow that combined computational vaccinology tools, laboratory analysis techniques, and sequence conservation analysis. In silico analysis to predict class II epitope clusters within PfCSP was performed using the PfCSP 3D7 vaccine strain and 539 PfCSP sequence variants as input sequences. Six class II epitope clusters were predicted in the PfCSP 3D7 sequence and ten class II epitope clusters (ICS) were predicted from the 539 PfCSP sequence variants. We downselected four clusters of interest from the PfCSP 3D7 sequence outputs, based on the number of predicted epitopes in the cluster, breadth of HLA-DRB1 alleles the epitopes were predicted to bind, and the cluster location. As two of the ten ICS overlapped PfCSP 3D7 sequences, the eight remaining ICS were downselected as of interest, all of which overlapped the TH2R/R2 region of PfCSP. This set of downselected clusters was further curated based on results of laboratory assays including *in vitro* assessments of HLA-DRB1 allele binding and *ex vivo* assessments of CD4 T cell recall responses, the latter resulting in multifunctional cytokine profiles characterized by increased IFN-γ, TNF-α, and IL-2 production and relatively little IL-4 and IL-10 production. We prioritized those class II epitope clusters capable of simulating multifunctional T cell responses for IFN-γ, TNF-α and/or IL-2, as a correlation between vaccine-induced sterile protection and induction of multifunctional T cells expressing high amounts of these cytokines has been demonstrated (41) and T cells simultaneously secreting more than one cytokine provide optimal effector function (42). Moreover, this basis for prioritization aligns with the finding that protected RTS,S vaccinees have a significantly higher frequency of multifunctional cytokine CD4 T cell responses as compared to unprotected vaccinees (4). With regard to final downselection, the clusters of most interest were evaluated for epitope conservation across 478 PfCSP sequence variants to ensure that final class II epitope cluster selection reflected the sequences most capable of eliciting cognate T cell help across PfCSP strains. The final selectees represent five variants of the PfCSP TH2R domain that have been shown to prime DC and T cell cultures, elicit multifunctional Th1 cytokine responses, and reflect a sequence set with epitope conservation across a large number of PfCSP variants. Notably, three of these final selectees (C3/ICS8, ICS7, and ICS9) were identified in the early *ex vivo* T cell simulation studies using PBMCs from malaria-exposed individuals (8).

Several aspects of this analysis strongly suggest that CSP is under immune pressure with regard to generation of cognate T cell help populations. Overall, based on size of this protein, the number of predicted class II T cell epitopes within PfCSP 3D7 is fairly low. This is indicated by relatively low overall class II epitope EpiMatrix score of -20 for the protein (proteins with overall EpiMatrix scores of +20 and higher are considered to have strong immune potential). Further, the conserved PfCSP class II T cell epitope clusters that showed good immune potential, based on in silico analysis (C2 and C4), demonstrated the most limited *ex vivo* responses across the broad set of HLA alleles represented among the 30 donors. However, the most variable epitope clusters C3 and TH2R/R2 variants generally demonstrated the broadest responses across the 30 donors. In the human host, elicitation of poor/limited T cell responses to conserved regions of PfCSP is of advantage to the parasite as an immune evasion strategy. Based on this, and in consideration of the in silico analysis, *ex vivo* studies, and predicted strain coverage, we recommend five ICS for CSP vaccine development (C3/ICS8, ICS2, ICS5, ICS7, and ICS9) to expand the available cognate T cell help in a manner that facilitates cross-strain coverage.

The primary T cell subset that provides cognate T help and drives antibody responses is Tfh, which play a central role in B cell affinity maturation, isotype switching and memory (20). Further, robustness and longevity of such humoral responses are regulated by the interplay between Tfh and Tfr (21, 22). Based on this and the relatively short-lived humoral responses seen with RTS,S (1), class II epitope clusters with a high potential to activate Treg should be circumvented in order to best optimize humoral responses. To this end, our workflow included assessments using the JanusMatrix tool to identify cross-conservation of the identified epitope clusters with the human proteome as a means to flag Treg cell activation potential. Further, our *ex vivo* CD4 T cell simulation assessments found that a relatively low percentage of donors produced IL-10, suggesting minimal Treg cell activation.

In addition to avoiding vaccine antigens containing sequences with cross-conservation to the human proteome, other strategies to modulate Treg suppression during vaccination are actively being researched and include efforts to develop novel adjuvants that induce effector T cells while modulating Treg activity or recruitment (43–45). For example, targeting C-C chemokine receptor 4 expression on Treg cells, in experimental models, enhanced T and B cell responses using various antigens (43, 45). Further, as programmed cell death (PD1) expression in Treg cells is indispensable for their suppressive functions (46) and PD1 upregulation upon naïve T cell activation plays a regulatory role in naïve-to-effector T cell differentiation (47), modulation of PD1 signaling may increase vaccine induced, antigen-specific responses. In this regard, we have recently demonstrated that when a peptide-based PD1 antagonist is prophylactically-combined with an adenovirus-based or irradiated sporozoite-based malaria vaccination, antigen-specific CD8 T cell expansion is enhanced (48). Additionally, with the same peptide-based PD1 antagonist, we found that therapeutic treatment of mice infected with a lethal malaria strain resulted in survival that was associated with lower numbers of Treg cells (48). When taken together with the data suggesting poor immune response to malaria infection is partially due to Treg cell activation and that interference in regulation of immune checkpoint proteins can lead to chronic malaria disease (24–26), we posit the need for minimizing epitopes cross-conserved with the human proteome and the inclusion of mechanisms to boost immune response through modulation of checkpoint proteins such as PD1 in best practice PfCSP vaccine development strategies. Moreover, we suggest such strategies are particularly important based on the relatively short-lived humoral responses found in RTS,S vaccinees and to overcome possible immune dysfunction (e.g., T cell exhaustion) in vaccinees with past malaria exposure.

While the focus of this work centered on identification of PfCSP class II epitope clusters, we recognize the possibility that elicitation of CD8 T cell responses to PfCSP epitopes may be relevant based on vaccine platform and/or in multi-antigen vaccine context. To this end, the prediction and evaluation of class I HLA-A and HLA-B epitopes was included as part of our umbrella strategy for PfCSP vaccine development. Our work in this area utilized the PfCSP 3D7 vaccine strain and 539 PfCSP sequence variants as input sequences for in silico epitope prediction across a panel of six class I HLA-A and HLA-B alleles (A*0101, A*0201, A*0301, A*2402, B*0702, B*4402) and included assessment of *in vitro* HLA binding *via* a competition assay format (see **Table S11** for reference peptide sequences) to 77 of the predicted epitopes synthesized as peptides (**Table S12**). Overall, 58% of the predicted class I epitopes that were tested bound the class I HLA allele (*in vitro*) that they were predicted to bind (**Table S13**). Only three of the predicted class I HLA epitopes were assessed for CD8 T cell response stimulation in the MIMIC platform. While all three of these sequences were previously shown to be determinants of CD8 T cell responses (49–51), only one demonstrated strong cytokine recall responses across multiple donors (**Table S14**) and these responses were multifunctional for IFN-γ, TNF-α, and IL-2 **(** **Table S15**). Notably, this epitope overlaps the PfCSP 3D7 TH2R region.

In consideration of future vaccine construct development with the downselected ICS, selection of an appropriate vaccine platform is critical. The sequence and structure of the TH2R/R2 domain variants may complicate vaccine development due to the presence of hydrophobic residues and the secondary structure; this region forms an alpha helix that interacts with and/or is stabilized by two beta sheets (52). Therefore, due consideration of the sequences for inclusion in a PfCSP vaccine should inform platform selection. Further, inclusion of B cell epitopes is critical in CSP vaccines where expanding the cognate T help repertoire is intended to increase protective antibodies in a manner that provides efficacy when vaccinees are challenged/infected with either homologous or heterologous PfCSP strains. While identifying the optimal composition of the B cell epitopes for inclusion in a PfCSP vaccine is outside the scope of work detailed herein, we recommend that this also be taken into account when selecting a vaccine platform. Lastly, although vaccine construct development and evaluation of *ex vivo* T cell response profiles with these sequences as part of a vaccine construct was also outside of our scope of work, we highly recommend such studies (using human PBMCs) prior to advanced PfCSP vaccine development.

## Data Availability Statement

The original contributions presented in the study are included in the article/**Supplementary Material**. Further inquiries can be directed to the corresponding author.

## Ethics Statement

The studies involving human participants were reviewed and approved by Advarra, Protocol CRRI 0906009. The patients/participants provided their written informed consent to participate in this study.

## Author Contributions

Overall conceptualization and study designs were contributed by AN, KT, JC, TP, VK, and GG. *In silico* analyses and *in vitro* HLA binding assays were performed by FT, LM, and PH, with supervision by WM and ADG. *Ex vivo* lymphocyte activation assays were performed and formally analysed by BS and ES, with supervision by DD. Project management, data organization, and formal analyses were performed by AN. Manuscript writing was performed by AN, TP, and GG. Manuscript editing and review were performed by FT, PH, LM, ADG, and BS. All authors contributed to the article and approved the submitted version.

## Funding

These studies were made possible through support provided by the Office of Infectious Diseases, Bureau for Global Health, U.S. Agency for International Development (https://www.usaid.gov), under the terms of the Malaria Vaccine Development Program (MVDP) Contract AID-OAA-C-15-00071, for which Leidos, Inc. is the prime contractor. The opinions expressed herein are those of the authors and do not necessarily reflect the views of the U.S. Agency for International Development. The funders approved study plans but had no direct role in development of study designs, data collection/analysis, or preparation of the manuscript.

## Conflict of Interest

AN, TP, KT, VK, JC, and GG are employees of Leidos, Inc., prime contractor for USAID Malaria Vaccine Development Program (MVDP) Contract AID-OAA-C-15-00071, and hold Leidos stock and/or stock options. FT, LM, WM, and ADG are employees of EpiVax, Inc., an MVDP subcontractor. PH was a previous employee of EpiVax, Inc. BS, ES, and DD are employees of Sanofi Pasteur, an MVDP subcontractor, and hold Sanofi Pasteur stock and/or stock options.
