# Supplementary material for: Bridging Computational Vaccinology and Vaccine Development Through Systematic Identification, Characterization, and Downselection of Conserved and Variable Circumsporozoite Protein CD4 T Cell Epitopes From Diverse Plasmodium falciparum Strains
Source: Front Immunol. 2021 Jun 8;12:689920. doi: 10.3389/fimmu.2021.689920 (PMC8217813; doi:10.3389/fimmu.2021.689920)
Supplement: Supplementary file 1 [file DataSheet_1.docx]

Supplementary Material

# Overview

This supplement includes data and information regarding both the PfCSP class II epitope identification and analysis (**Section 3**) and the PfCSP class I epitope identification and analysis (**Section 4**). With regard to the latter, these data were not the primary focus of our PfCSP epitope-directed vaccine design efforts but rather were included in our overall vaccine strategy as a bridge to multi-antigen vaccine development efforts directed at the sporozoite and liver stages of the parasite. We have provided the PfCSP class I epitope data herein to make these available to the broader research community.

# Table of Contents

[1. Overview 1](#_Toc71211309)

[2. Table of Contents 1](#_Toc71211310)

[3. PfCSP Class II Epitope Prediction and Analysis 3](#_Toc71211311)

[Supplementary Figure 1. Sequence Coverage for the PfCSP Variant Sequence Database. 3](#_Toc71211312)

[Supplementary Figure 2. Geographic Origin and Distribution of the 539 PfCSP Sequence Variants. 4](#_Toc71211313)

[Supplementary Table 1. Maximum EpiMatrix Z Score and Significant Z Score Count for all Predicted PfCSP Class II T Cell Epitope Clusters. 5](#_Toc71211314)

[Supplementary Figure 3. PfCSP 3D7 EpiMatrix Cluster Scores Relative to Known Promiscuous Epitopes. 6](#_Toc71211315)

[Supplementary Table 2. Predicted Class II Epitope Cross-Conservation with the Human Proteome as a Measure of Regulatory T Cell Response Potential. 7](#_Toc71211316)

[Supplementary Table 3. PfCSP 3D7 Cross-Conservation Sequence Analysis with 539 PfCSP Sequence Variants. 8](#_Toc71211317)

[Supplementary Table 4. Reference Peptide Sequences Utilized for the In Vitro Class II HLA Allele Binding Assay. 9](#_Toc71211318)

[Supplementary Table 5. PfCSP Epitope In Vitro Binding Affinity (as IC50 in nM) for Class II HLA Alleles. 10](#_Toc71211319)

[Supplementary Table 6. Class II Epitope In Silico Predictions Demonstrated an Accuracy of 79% in Predicting Epitopes that Bind HLA-DR Alleles In Vitro. 11](#_Toc71211320)

[Supplementary Figure 4. Representative Flow Cytometry Data for a Single Donor Illustrating CD4+CD154+IFN-γ+ Cytokine Responses. 12](#_Toc71211321)

[Supplementary Table 7. Percentage of Donors by HLA Type Demonstrating TNF-α Responses to Peptides Comprised of PfCSP Predicted Epitope Clusters. 13](#_Toc71211322)

[Supplementary Table 8. Percentage of Donors by HLA Type Demonstrating IL-2 Responses to Peptides Comprised of PfCSP Predicted Epitope Clusters. 14](#_Toc71211323)

[Supplementary Table 9. Percentage of Donors by HLA Type Demonstrating IL-4 Responses to Peptides Comprised of PfCSP Predicted Epitope Clusters. 15](#_Toc71211324)

[Supplementary Table 10. Percentage of Donors by HLA Type Demonstrating IL-10 Responses to Peptides Comprised of PfCSP Predicted Epitope Clusters. 16](#_Toc71211325)

[4. PfCSP Class I Epitope Prediction and Analysis 17](#_Toc71211326)

[Supplementary Materials and Methods: Class I Assessments 17](#_Toc71211327)

[Supplementary Table 11. Reference and Negative Control Peptide Sequences Utilized for the In Vitro Class I HLA Allele Binding Assay. 18](#_Toc71211328)

[Supplementary Table 12. Class I HLA Allele In Vitro Binding Assay Data. 19](#_Toc71211329)

[Supplementary Table 13. Peptide Binding Rates for Class I HLA Alleles. 21](#_Toc71211330)

[Supplementary Table 14. Class I PfCSP 3D7 (PFC0210c)) Epitopes Assessed by VaxDesign and the Number of Donor Responses (by HLA haplotype) with a Positive SI (SI≥2.0) 22](#_Toc71211331)

[Supplementary Table 15. Functional CD8 T Cell Response Profiles for Class I PfCSP 3D7 Epitopes Assessed by VaxDesign 22](#_Toc71211332)

# PfCSP Class II Epitope Prediction and Analysis





Supplementary Figure 1. Sequence Coverage for the PfCSP Variant Sequence Database.

A total of 539 publicly available PfCSP sequence variants were compiled for analysis. While all of the sequences were used to generate the EpiAssembler output, only 478 of these sequence variants contained the TH2R/R2 region of PfCSP. Regions of PfCSP are shown in the top bar as follows: signal sequence (first grey box), R1 (first blue box), repeats (second grey box), TH2R (first red box), R2 (second blue box), and TH3R (second red box). For the sequences, white spaces represent sequence gaps. Conserved and variable regions of CSP can be seen based on uniform and non-uniform vertical patterning, respectively.


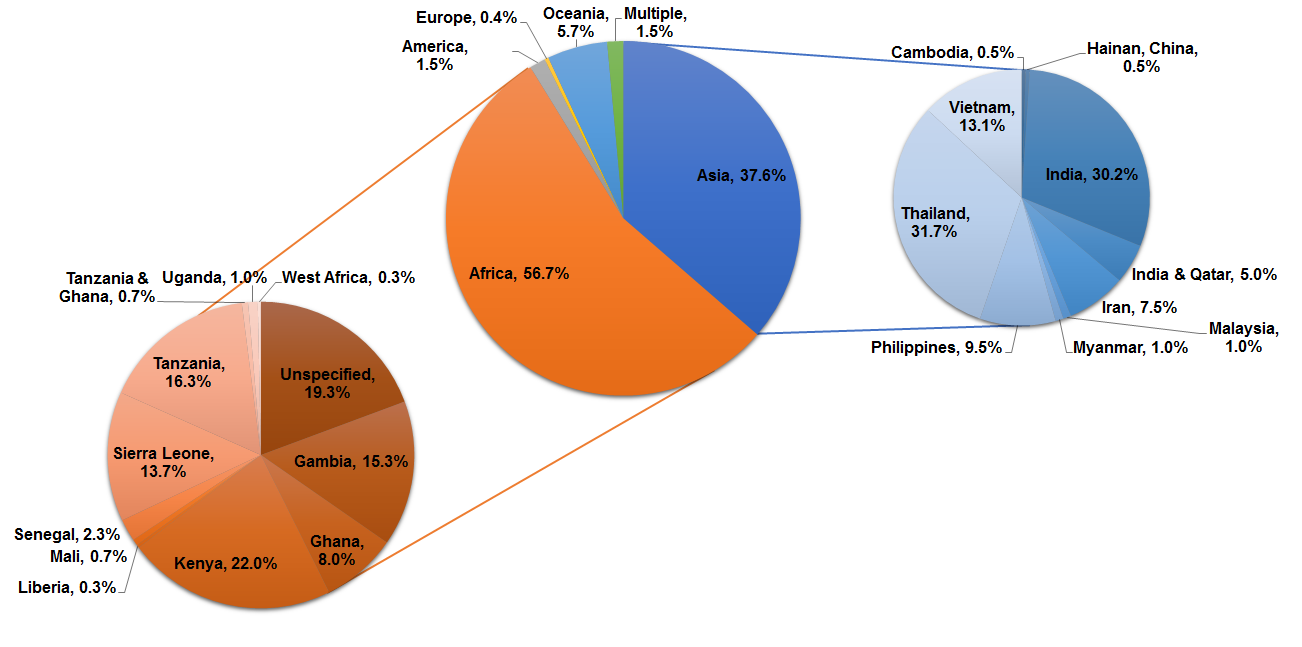


Supplementary Figure 2. Geographic Origin and Distribution of the 539 PfCSP Sequence Variants.

Supplementary Table 1. Maximum EpiMatrix Z Score and Significant Z Score Count for all Predicted PfCSP Class II T Cell Epitope Clusters.

EpiMatrix output from iVAX toolkit showing the highest Z score and total number of predicted epitopes (EpiMatrix hit count) for all PfCSP class II clusters across the set of HLA-DR alleles evaluated. EpiMatrix output identifies highly significant Z scores, in the top 1% (>2.31) and significant Z scores, in the top 5% (>1.63). Color coding indicates if the highest Z score falls in the top 1% (noted in dark blue) or the top 5% (noted in light blue). EpiMatrix hit counts for each HLA-DR are shown in parentheses. Predicted clusters shown are based on the ClustiMer output.

|  | **DRB1*0101** | **DRB1*0301** | **DRB1*0401** | **DRB1*0701** | **DRB1*0801** | **DRB1*0901** | **DRB1*1101** | **DRB1*1301** | **DRB1*1501** |
| --- | --- | --- | --- | --- | --- | --- | --- | --- | --- |
| **Cluster ID** | **EPX Max Z (Total Hits)** | **EPX Max Z (Total Hits)** | **EPX Max Z (Total Hits)** | **EPX Max Z (Total Hits)** | **EPX Max Z (Total Hits)** | **EPX Max Z (Total Hits)** | **EPX Max Z (Total Hits)** | **EPX Max Z (Total Hits)** | **EPX Max Z (Total Hits)** |
| **SS1** | **1.95 (2)** | **1.91 (3)** | **2.14 (1)** | **2.71 (4)** | **2.37 (1)** | **2.57 (3)** | **2.18 (3)** | **2.29 (2)** | **2.51 (4)** |
| **SS2** | **2.25 (1)** | **1.86 (2)** | **2.75 (1)** | **2.55 (1)** | **2.03 (1)** | **2.04 (1)** | **2.85 (1)** | **2.04 (1)** | **2.34 (1)** |
| **C1** | **2.16 (1)** | **1.70 (1)** | **1.87 (1)** | **2.33 (2)** | **1.82 (1)** | **2.44 (1)** | **1.18 (0)** | **1.34 (0)** | **1.72 (1)** |
| **C2** | **2.02 (4)** | **2.69 (3)** | **2.00 (2)** | **1.84 (2)** | **3.31 (3)** | **1.96 (1)** | **3.02 (4)** | **2.55 (3)** | **1.94 (1)** |
| **C’** | **1.26 (0)** | **1.50 (0)** | **0.98 (0)** | **1.94 (1)** | **2.03 (4)** | **0.99 (0)** | **1.88 (1)** | **2.28 (1)** | **2.19 (1)** |
| **C3/ICS8** | **2.59 (2)** | **2.02 (1)** | **2.92 (3)** | **2.58 (5)** | **2.20 (2)** | **2.36 (3)** | **2.52 (4)** | **2.23 (2)** | **2.62 (3)** |
| **C4/ICS1** | **2.34 (4)** | **2.27 (5)** | **2.32 (2)** | **2.55 (7)** | **2.12 (1)** | **2.54 (7)** | **2.18 (2)** | **2.04 (4)** | **2.43 (5)** |
| **ICS2** | **2.79 (3)** | **2.23 (2)** | **3.12 (3)** | **2.67 (4)** | **2.18 (2)** | **2.36 (3)** | **2.74 (3)** | **2.44 (1)** | **2.82 (2)** |
| **ICS3** | **2.63 (2)** | **2.06 (2)** | **2.96 (2)** | **2.51 (4)** | **2.20 (2)** | **2.35 (3)** | **2.56 (2)** | **2.27 (2)** | **2.66 (2)** |
| **ICS4** | **2.40 (3)** | **1.84 (1)** | **2.74 (3)** | **3.01 (2)** | **1.94 (1)** | **2.61 (3)** | **2.34 (1)** | **2.70 (2)** | **2.44 (2)** |
| **ICS5** | **2.78 (3)** | **2.22 (2)** | **3.10 (4)** | **2.95 (4)** | **2.31 (3)** | **2.54 (3)** | **2.72 (4)** | **2.42 (2)** | **2.81 (3)** |
| **ICS6** | **2.74 (1)** | **2.76 (3)** | **3.06 (3)** | **2.59 (4)** | **3.10 (4)** | **2.54 (3)** | **2.68 (4)** | **2.39 (3)** | **2.77 (2)** |
| **ICS7** | **2.38 (2)** | **1.50 (0)** | **1.95 (2)** | **2.44 (4)** | **2.20 (2)** | **2.25 (4)** | **1.78 (3)** | **1.89 (1)** | **2.25 (2)** |
| **ICS9** | **2.88 (3)** | **2.33 (1)** | **3.21 (3)** | **2.54 (4)** | **2.15 (3)** | **2.36 (3)** | **2.83 (3)** | **2.53 (2)** | **2.91 (2)** |
| **ICS10** | **2.51 (2)** | **2.05 (3)** | **2.85 (2)** | **2.66 (4)** | **2.55 (2)** | **2.84 (3)** | **2.45 (2)** | **2.16 (3)** | **2.55 (2)** |


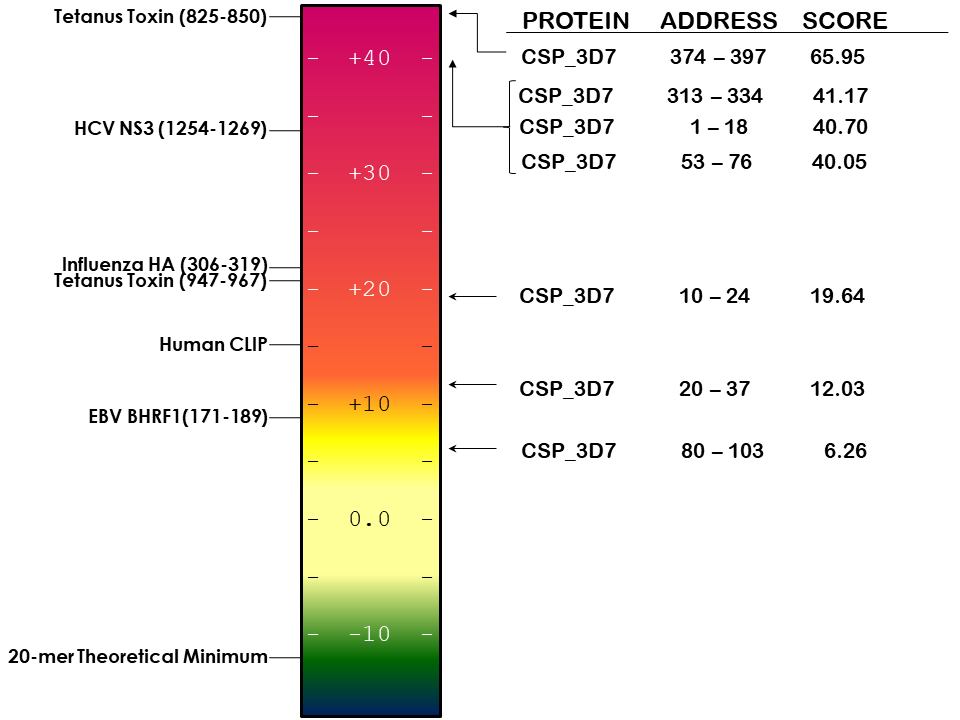


Supplementary Figure 3. PfCSP 3D7 EpiMatrix Cluster Scores Relative to Known Promiscuous Epitopes.

Known promiscuous class II epitopes have EpiMatrix cluster scores above ten (shown on left). The six PfCSP 3D7 clusters have EpiMatrix cluster scores above ten; however, the pseudo-cluster (80-103) does not reach this threshold (shown on right).

Supplementary Table 2. Predicted Class II Epitope Cross-Conservation with the Human Proteome as a Measure of Regulatory T Cell Response Potential.

EpiMatrix (EPX) cluster scores (a measure of immune potential) and JanusMatrix (JMX) cluster scores (a measure of humanness, e.g., immunosuppression potential) are shown for the clusters of in silico predicted class II epitopes within PfCSP 3D7 (above the triple line) and PfCSP sequence variants for the TH2R/R2 region (below the triple line). A JMX cluster score above 2.00 denotes an increase potential for immunosuppressive (e.g., Treg) responses. The number of human matches indicates the number of human proteins containing synonymous HLA-DR binding frames with matching TCR-binding face residues as compared those in the PfCSP clusters. For each HLA-DR allele, the match count is shown (i.e., total number of Z scores ≥1.64 for each allele from the JMX algorithm output). Peptides trimmed from the original in silico output are noted with trimmed amino acids shown in red font.

| **Cluster ID** | **Sequence** | **EPX Cluster Score** | **Number HUMAN Matches** | **JMX Cluster Score** | **JMX Total Z Score Hit Count (≥1.64)** | | | | | | | | |
| --- | --- | --- | --- | --- | --- | --- | --- | --- | --- | --- | --- | --- | --- |
|  |  |  |  |  | **DRB1 *0101** | **DRB1 *0301** | **DRB1 *0401** | **DRB1 *0701** | **DRB1 *0801** | **DRB1 *0901** | **DRB1 *1101** | **DRB1 *1301** | **DRB1 *1501** |
| SS1 | MMRKLAILSVSSFLFVEA | 40.7 | 40 | 3.57 | 12 | 2 | 5 | 10 | 2 | 9 | 11 | 4 | 28 |
| SS2 | VSSFLFVEALFQEYQ | 19.64 | 4 | 0.90 | 1 | 3 | 1 | 1 | 0 | 1 | 0 | 1 | 1 |
| C1 | FQEYQCYGSSSNTRVLNE | 12.03 | 1 | 0.13 | 0 | 0 | 1 | 0 | 0 | 0 | 0 | 0 | 0 |
| C2 | MNYYGKQENWYSLKKNSRSLGEND | 40.05 | 25 | 2.59 | 8 | 8 | 3 | 6 | 11 | 0 | 5 | 10 | 6 |
| C' | NEDNEKLRKPKHKKLKQPADGNPD | 6.26 | 27 | 3.88 | 0 | 0 | 0 | 8 | 10 | 0 | 0 | 11 | 2 |
| C3/ICS8 | DKHIKEYLNKIQNSLSTEWSPC | 41.17 | 20 | 2.00 | 3 | 1 | 5 | 10 | 6 | 3 | 8 | 7 | 7 |
| C4/ICS1 | CSSVFNVVNSSIGLIMVLSFLFLN ^a^ | 65.95 | 19 | 1.22 | 3(2) | 5(5) | 2(0) | 10(7) | 0(0) | 13(6) | 2(0) | 2(2) | 8(2) |
| ICS2 | ITDYLKKIQNSLSTEWSPCS | 45.19 | 31 | 4.00 | 10 | 13 | 9 | 15 | 8 | 10 | 14 | 5 | 8 |
| ICS3 | DQHIEQYLKKIQNSISTEWS | 35.01 | 18 | 2.24 | 3 | 12 | 4 | 10 | 3 | 6 | 2 | 5 | 2 |
| ICS4 | DQHIEQYLKTIQNSLSTEWS | 27.15 | 21 | 2.39 | 9 | 3 | 5 | 7 | 2 | 7 | 1 | 4 | 5 |
| ICS5 | DQHIEKYLKIIQNSLSTEWSP | 47.79 | 24 | 1.89 | 5 | 1 | 13 | 11 | 3 | 7 | 8 | 1 | 4 |
| ICS6 | IKKYLKKIKNSISTEWSPCS | 41.12 | 37 | 2.93 | 2 | 12 | 5 | 13 | 16 | 7 | 12 | 7 | 5 |
| ICS7 | IEQYLKKIQYSLSTEWSPC | 27.04 | 16 | 2.10 | 6 | 0 | 6 | 8 | 1 | 5 | 7 | 0 | 8 |
| ICS9 | DKHIEKYLKRIQNSLSTEWS | 41.67 | 26 | 2.79 | 10 | 2 | 10 | 13 | 10 | 8 | 7 | 3 | 5 |
| ICS10 | DQHIEKYLKTIKNSLSTEWS | 36.14 | 20 | 2.35 | 4 | 11 | 5 | 10 | 4 | 8 | 6 | 4 | 5 |

^a^ To facilitate synthesis and reconstitution, the C4/ICS1 peptide was trimmed from original ClustiMer algorithm output sequence. Some of the JanusMatrix hits for this sequence overlapped the C-terminal residues that were trimmed; therefore, an adjusted hit count for the trimmed sequence is provided in parenthesis.

Supplementary Table 3. PfCSP 3D7 Cross-Conservation Sequence Analysis with 539 PfCSP Sequence Variants.

The cross-conservation of predicted class II epitope clusters was evaluated comparing sequences found in PfCSP 3D7 to those of 539 PfCSP sequence variants. Clusters of interest in the N-terminal region of PfCSP (i.e., those C-terminal of the signal sequence) were highly conserved with >80% cross-conservation. Clusters in the C-terminal region of PfCSP were less well conserved. In particular, the sequences overlapping the TH2R/R2 region only had 19% cross-conservation with the C3 sequence from PfCSP 3D7. Of note is that although the SS1, SS2, and C’ clusters were included in this analysis for informational purposes, these clusters were not downselected for further evaluation based on location (for SS1 and SS2) and EpiMatrix/JanusMatrix cluster scores (for C’).

| **Cluster Address ^a^** | **PfCSP 3D7 Matched Cluster ID** | **EpiMatrix Cluster Score** | **Cluster Homology Score** | **Cluster Cross Conservation** |
| --- | --- | --- | --- | --- |
| 1 – 18 | SS1 | 40.70 | 184 | 56% ^b^ |
| 10 – 24 | SS2 | 19.64 | 224 | 68% ^b^ |
| 20 – 37 | C1 | 12.03 | 272 | 83% ^b^ |
| 53 – 76 | C2 | 40.05 | 280 | 85% ^b^ |
| 80 – 103 | C’ | 6.26 | 271 | 82% ^b^ |
| 313 – 334 | C3 | 41.17 | 101 | 19% ^c^ |
| 374 – 397 | C4 | 65.95 | 323 | 62% ^c^ |

^a^ Cluster addresses were normalized to the PfCSP 3D7 sequence

^b^ A total of 329 PfCSP sequence variants included amino acids in the N-terminal region of CSP

^c^ A total of 525 PfCSP sequence variants included amino acids in the N-terminal region of CSP

Supplementary Table 4. Reference Peptide Sequences Utilized for the In Vitro Class II HLA Allele Binding Assay.

| **Allele** | **Reference Peptide Sequence** |
| --- | --- |
| **DRB1*0101** | **PRYVKQNTLKLAT** |
| **DRB1*0303** | **LFRKDIAAKYKE** |
| **DRB1*0401** | **YARFQSQTTLKQKT** |
| **DRB1*0701** | **QYIKANSKFIGITEL** |
| **DRB1*0801** | **KLYRKLKREITFH** |
| **DRB1*1101** | **QYIKANSKFIGITEL** |
| **DRB1*1301** | **IELGKKFNIKTRLSYFSMNH** |
| **DRB1*1501** | **NPVVHFFKNIVTPRTPPPS** |
| **Negative control** | **ISQAVHAAHAEINEAGR** |

Supplementary Table 5. PfCSP Epitope In Vitro Binding Affinity (as IC50 in nM) for Class II HLA Alleles.

A subset of predicted PfCSP class II epitope clusters were synthesized as peptides and assessed for HLA-DR binding in vitro utilizing a binding inhibition assay.

|  |  | **DRB1*0101** | **DRB1*0301** | **DRB1*0401** | **DRB1*0701** | **DRB1*0801** | **DRB1*1101** | **DRB1*1301** | **DRB1*1501** |
| --- | --- | --- | --- | --- | --- | --- | --- | --- | --- |
| **Cluster ID** | **Peptide Sequence** | **IC_50_** | **IC_50_** | **IC_50_** | **IC_50_** | **IC_50_** | **IC_50_** | **IC_50_** | **IC_50_** |
| C1 | KKFQEYQCYGSSSNTRVLNE-amide^b^ | **50142** | **205836** | **176369** | **36664** | **8.E+08** | **6.E+09** | **9.E+08** | **1767** |
| C2 | Ac-KQENWYSLKKNSRSLGEND-amide^a^ | **115500** | **138248** | **10834** | **131230** | **108** | **395** | **37** | **302422** |
| C3/ICS8 | Ac-KHIKEYLNKIQNSLSTEWS-amide^a^ | **572** | **107131** | **2003** | **2375** | **144** | **16350** | **11961** | **74** |
| C4/ICS1 | KSSVFNVVNSSIGLIMVLS-amide^a,b^ | **526** | **1095** | **1690** | **2072** | **1.E+09** | **1040** | **1204** | **12325** |
| ICS2 | Ac-ITEYLKKIQNSLSTEWSPCS-amide^c^ | **83940** | **1.E+07** | **377** | **20022** | **4760** | **43671** | **73233** | **8470** |
| ICS5 | Ac-DQHIEKYLKIIQNSLSTEWSP-amide | **218** | **84661** | **35** | **3684** | **23114** | **5555** | **19784** | **30** |
| ICS7 | Ac-IEQYLKKIQYSLSTEWSPC-amide | **Non-binder** | **2.E+09** | **32763** | **2761** | **6089** | **1741993** | **17934** | **63249** |
|  | | | | | | | | | |
|  | | **Class II HLA Allele IC_50_ Binding Affinity Color Key** | | | |  | | | |
|  | | **Very High Affinity (IC_50_ < 100nM)** | | | |  | | | |
|  | | **High Affinity (100 nM < IC_50_ < 1,000 nM)** | | | |  | | | |
|  | | **Moderate Affinity (1,000 nM < IC_50_ < 10,000 nM)** | | | |  | | | |
|  | | **Low Affinity (10,000 nM < IC_50_ < 100,000 nM)** | | | |  | | | |
|  | | **Negligible Affinity (100,000 nM < IC_50_)** | | | |  | | | |
|  | | **Non-binder (No dose-dependent inhibition)** | | | |  | | | |
| ^a^ Peptide trimmed from original ClustiMer algorithm output sequence.  ^b^ Charge added to facilitate peptide synthesis and purification; shown in red.  ^c^ D to E PfCSP sequence variant used to reduce JanusMatrix peptide score from 4.00 to 3.77 without impact to EpiMatrix peptide score; shown in red. Note this change is in a flanking residue, not within the binding core. | | | | | | | | | |

Supplementary Table 6. Class II Epitope In Silico Predictions Demonstrated an Accuracy of 79% in Predicting Epitopes that Bind HLA-DR Alleles In Vitro.

The Z-score cut-off for the EpiMatrix algorithm is configured to include some peptides with lower binding affinities (increased sensitivity) in order to reduce the possibility of missing high-quality targets (type 2 error). This strategy results in a relatively high false positive rate (reduced specificity). Accuracy is calculated as the number of correct assessments divided by the number of all assessments or Accuracy = (TN + TP)/(TN+TP+FN+FP).

|  |  | **In Silico HLA-DR Binding Prediction  (EpiMatrix Scores ≥ 1.64)** | |
| --- | --- | --- | --- |
|  |  | **Positive** | **Negative** |
| **In Vitro HLA-DR Allele Binding (IC_50_ < 100,000 nM)** | **Positive** | **41  (True Positive)** | **0  (False Negative)** |
|  | **Negative** | **12  (False Positive)** | **3  (True Negative)** |
|  |  | **Accuracy = 79%** | |


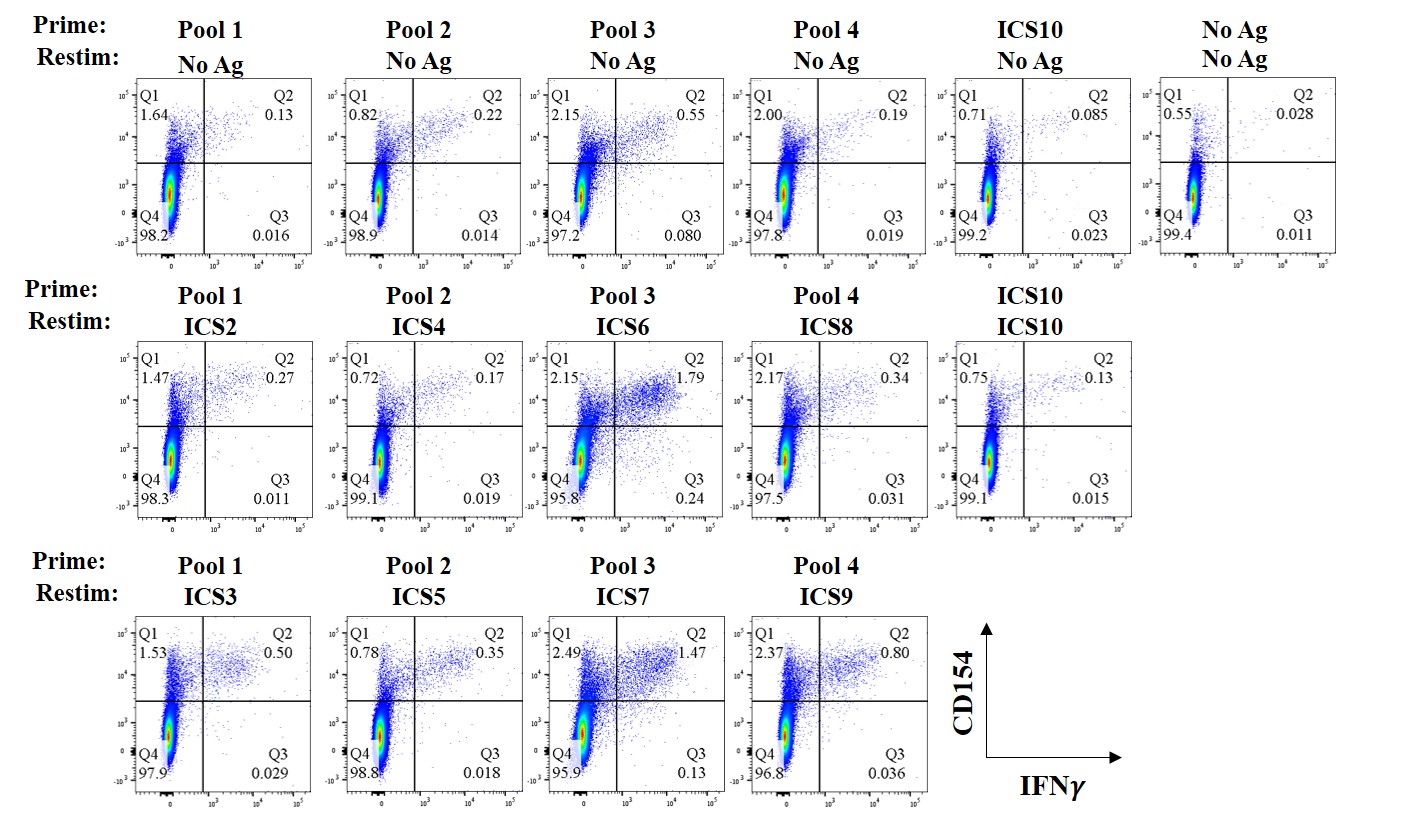


Supplementary Figure 4. Representative Flow Cytometry Data for a Single Donor Illustrating CD4+CD154+IFN-γ+ Cytokine Responses.

Flow cytometry plots from a representative donor illustrating the typical CD4+CD154+IFN-γ+ cytokine responses following a short restimulation assay with each ICS peptide. The full matrix presented with co-culture priming condition is listed above the restimulation (restim) condition. Priming pools were established with two peptides per pool. Primed cultures were divided and restimulated individually with untreated DCs (No Ag) or peptide-primed DCs corresponding to the associated priming pool.

Supplementary Table 7. Percentage of Donors by HLA Type Demonstrating TNF-α Responses to Peptides Comprised of PfCSP Predicted Epitope Clusters.

A Stimulation Index greater or equal to 1.5-fold above baseline is used to specify a positive donor response. The percentage of responding donors, by HLA allele for each predicted 3D7 and ICS cluster, is shown numerically along with color-coding to indicate higher percentages of responders with darker blues. The number of HLA-matched donors (N) for each allele is shown in the bottom row. A total of 30 donors were included in this study.

| **TNF-α** | **HLA Allele** | | | | | | | | |
| --- | --- | --- | --- | --- | --- | --- | --- | --- | --- |
| **Cluster ID** | **DR1** | **DR3** | **DR4** | **DR7** | **DR8** | **DR9** | **DR11** | **DR13** | **DR15** |
| **C2** | **0%** | **0%** | **0%** | **0%** | **0%** | **0%** | **0%** | **0%** | **0%** |
| **C3/ICS8** | **40%** | **33%** | **40%** | **50%** | **50%** | **50%** | **75%** | **14%** | **14%** |
| **C4** | **0%** | **0%** | **10%** | **0%** | **25%** | **25%** | **25%** | **43%** | **14%** |
| **ICS2** | **40%** | **17%** | **50%** | **25%** | **50%** | **25%** | **50%** | **71%** | **29%** |
| **ICS3** | **60%** | **0%** | **40%** | **50%** | **25%** | **25%** | **50%** | **14%** | **29%** |
| **ICS4** | **0%** | **0%** | **30%** | **0%** | **0%** | **0%** | **0%** | **0%** | **29%** |
| **ICS5** | **40%** | **17%** | **40%** | **0%** | **75%** | **50%** | **75%** | **43%** | **29%** |
| **ICS6** | **20%** | **33%** | **30%** | **25%** | **0%** | **50%** | **50%** | **43%** | **43%** |
| **ICS7** | **40%** | **50%** | **80%** | **75%** | **100%** | **50%** | **25%** | **71%** | **57%** |
| **ICS9** | **40%** | **50%** | **70%** | **50%** | **100%** | **50%** | **75%** | **57%** | **43%** |
| **ICS10** | **0%** | **17%** | **10%** | **25%** | **0%** | **25%** | **25%** | **14%** | **14%** |
| **N** | **5** | **6** | **10** | **4** | **4** | **4** | **4** | **7** | **7** |

Supplementary Table 8. Percentage of Donors by HLA Type Demonstrating IL-2 Responses to Peptides Comprised of PfCSP Predicted Epitope Clusters.

A Stimulation Index greater or equal to 1.5-fold above baseline is used to specify a positive donor response. The percentage of responding donors, by HLA allele for each predicted 3D7 and ICS cluster, is shown numerically along with color-coding to indicate higher percentages of responders with darker blues. The number of HLA-matched donors (N) for each allele is shown in the bottom row. A total of 30 donors were included in this study.

| **IL-2** | **HLA Allele** | | | | | | | | |
| --- | --- | --- | --- | --- | --- | --- | --- | --- | --- |
| **Cluster ID** | **DR1** | **DR3** | **DR4** | **DR7** | **DR8** | **DR9** | **DR11** | **DR13** | **DR15** |
| **C2** | **0%** | **0%** | **0%** | **0%** | **0%** | **0%** | **0%** | **0%** | **0%** |
| **C3/ICS8** | **20%** | **33%** | **40%** | **50%** | **50%** | **50%** | **25%** | **0%** | **14%** |
| **C4** | **0%** | **0%** | **0%** | **0%** | **25%** | **25%** | **25%** | **43%** | **0%** |
| **ICS2** | **40%** | **33%** | **20%** | **0%** | **25%** | **25%** | **25%** | **29%** | **14%** |
| **ICS3** | **80%** | **0%** | **40%** | **50%** | **0%** | **0%** | **25%** | **0%** | **14%** |
| **ICS4** | **20%** | **0%** | **20%** | **0%** | **0%** | **0%** | **25%** | **0%** | **14%** |
| **ICS5** | **20%** | **0%** | **40%** | **25%** | **0%** | **25%** | **50%** | **14%** | **0%** |
| **ICS6** | **20%** | **33%** | **30%** | **50%** | **0%** | **50%** | **25%** | **29%** | **29%** |
| **ICS7** | **60%** | **33%** | **70%** | **75%** | **75%** | **75%** | **25%** | **57%** | **57%** |
| **ICS9** | **20%** | **33%** | **50%** | **50%** | **75%** | **50%** | **50%** | **43%** | **43%** |
| **ICS10** | **0%** | **17%** | **30%** | **25%** | **0%** | **25%** | **50%** | **14%** | **14%** |
| **N** | **5** | **6** | **10** | **4** | **4** | **4** | **4** | **7** | **7** |

Supplementary Table 9. Percentage of Donors by HLA Type Demonstrating IL-4 Responses to Peptides Comprised of PfCSP Predicted Epitope Clusters.

A Stimulation Index greater or equal to 1.5-fold above baseline is used to specify a positive donor response. The percentage of responding donors, by HLA allele for each predicted 3D7 and ICS cluster, is shown numerically along with color-coding to indicate higher percentages of responders with darker blues. The number of HLA-matched donors (N) for each allele is shown in the bottom row. A total of 30 donors were included in this study.

| **IL-4** | **HLA Allele** | | | | | | | | |
| --- | --- | --- | --- | --- | --- | --- | --- | --- | --- |
| **Cluster ID** | **DR1** | **DR3** | **DR4** | **DR7** | **DR8** | **DR9** | **DR11** | **DR13** | **DR15** |
| **C2** | **40%** | **0%** | **10%** | **25%** | **0%** | **25%** | **0%** | **0%** | **0%** |
| **C3/ICS8** | **20%** | **0%** | **0%** | **0%** | **0%** | **0%** | **0%** | **0%** | **0%** |
| **C4** | **0%** | **0%** | **40%** | **25%** | **25%** | **25%** | **0%** | **14%** | **29%** |
| **ICS2** | **0%** | **33%** | **10%** | **0%** | **0%** | **0%** | **0%** | **14%** | **0%** |
| **ICS3** | **0%** | **17%** | **10%** | **0%** | **0%** | **0%** | **0%** | **0%** | **0%** |
| **ICS4** | **0%** | **17%** | **0%** | **0%** | **0%** | **0%** | **0%** | **14%** | **0%** |
| **ICS5** | **0%** | **33%** | **0%** | **25%** | **0%** | **0%** | **0%** | **14%** | **0%** |
| **ICS6** | **0%** | **0%** | **0%** | **0%** | **0%** | **25%** | **0%** | **0%** | **14%** |
| **ICS7** | **20%** | **0%** | **0%** | **0%** | **0%** | **0%** | **0%** | **0%** | **0%** |
| **ICS9** | **0%** | **33%** | **0%** | **25%** | **0%** | **0%** | **0%** | **0%** | **14%** |
| **ICS10** | **0%** | **17%** | **0%** | **0%** | **0%** | **0%** | **0%** | **0%** | **14%** |
| **N** | **5** | **6** | **10** | **4** | **4** | **4** | **4** | **7** | **7** |

Supplementary Table 10. Percentage of Donors by HLA Type Demonstrating IL-10 Responses to Peptides Comprised of PfCSP Predicted Epitope Clusters.

A Stimulation Index greater or equal to 1.5-fold above baseline is used to specify a positive donor response. The percentage of responding donors, by HLA allele for each predicted 3D7 and ICS cluster, is shown numerically along with color-coding to indicate higher percentages of responders with darker blues. The number of HLA-matched donors (N) for each allele is shown in the bottom row. A total of 30 donors were included in this study.

| **IL-10** | **HLA Allele** | | | | | | | | |
| --- | --- | --- | --- | --- | --- | --- | --- | --- | --- |
| **Cluster ID** | **DR1** | **DR3** | **DR4** | **DR7** | **DR8** | **DR9** | **DR11** | **DR13** | **DR15** |
| **C2** | **20%** | **50%** | **10%** | **50%** | **25%** | **25%** | **0%** | **29%** | **14%** |
| **C3/ICS8** | **0%** | **17%** | **10%** | **0%** | **25%** | **50%** | **0%** | **0%** | **29%** |
| **C4** | **20%** | **0%** | **20%** | **0%** | **25%** | **0%** | **0%** | **0%** | **0%** |
| **ICS2** | **0%** | **33%** | **0%** | **0%** | **25%** | **0%** | **0%** | **29%** | **14%** |
| **ICS3** | **20%** | **17%** | **0%** | **0%** | **0%** | **0%** | **0%** | **0%** | **14%** |
| **ICS4** | **0%** | **0%** | **10%** | **0%** | **0%** | **0%** | **0%** | **0%** | **0%** |
| **ICS5** | **0%** | **17%** | **0%** | **0%** | **0%** | **25%** | **0%** | **0%** | **14%** |
| **ICS6** | **20%** | **17%** | **30%** | **50%** | **0%** | **25%** | **25%** | **0%** | **43%** |
| **ICS7** | **0%** | **0%** | **10%** | **25%** | **0%** | **0%** | **0%** | **14%** | **14%** |
| **ICS9** | **0%** | **17%** | **10%** | **0%** | **0%** | **0%** | **0%** | **14%** | **43%** |
| **ICS10** | **0%** | **0%** | **10%** | **25%** | **0%** | **0%** | **0%** | **0%** | **0%** |
| **N** | **5** | **6** | **10** | **4** | **4** | **4** | **4** | **7** | **7** |

# PfCSP Class I Epitope Prediction and Analysis

Supplementary Materials and Methods: Class I Assessments

In Vitro Class I HLA-Peptide Analysis Binding Assay

The cell-free class I HLA binding assay in use at Pure Protein, LLC allows for in vitro quantification of peptide-HLA binding affinity in a competition format. In this assay, a fluorescently labeled, high binding, control peptide is loaded onto 384-well ½-area plates along with unlabeled experimental peptides. To remove endogenous peptides and render the HLA A and B molecules receptive to binding, HLA is heated at 53°C for 15 minutes and immediately added to the assay. The mixture is then allowed to stand for three days. Once the mixture has reached steady equilibrium (at 72 hours), displacement of the high binding control peptide is measured through Fluorescence Polarization. Binding of experimental peptides is expressed as the percent inhibition of the labeled control peptide (experimental fluorescence / control fluorescence, multiplied by 100). The percent inhibition values for each experimental peptide (across a range of molar concentrations) are used to calculate the concentration which inhibits 50% of the labeled control peptide’s specific binding. This value is referred to as the peptide’s IC50.

Ex Vivo MIMIC® CD8 T Cell Stimulation Assay

For this assay, PBMCs were collected as described in the materials and methods for the CD4 T cell stimulation assay. CD8 T cell stimulation assays were performed using protocols established at Sanofi Pasteur–VaxDesign Campus. CD8 T cells were sorted by negative selection using EasySep Human CD8 T cell isolation kit (Stemcell technologies). The resulting CD8 T cells (>95% pure) were plated at a concentration of 3x10^6^/mL in X-VIVO 15 media supplemented with 5ng/mL IL-7 overnight. CD8 T cells were then harvested and co-cultured with autologous dendritic cells (DCs) at a ratio of 60:1 in X-VIVO 15 media containing 30ng/mL of IL-21. The DCs were either untouched (control wells) or pre-pulsed for at least 2 hours with pooled peptides (each peptide at 5 µg/ml). On day 3 of the co-culture half of the culture media was replace with fresh X-VIVO 15 media containing IL-7 and IL-15 at a final concentration of 5ng/mL. On day 5 the co-culture was transferred from the original 48 well plate to a 12 well plate and 1mL of fresh X-VIVO 15 was added containing IL-7 and IL-15 at a final concentration of 5ng/mL. After a 12-day incubation period, lymphocytes were harvested and evaluated for effector activity using intracellular cytokine staining (ICCS). For the ICCS, autologous DCs were pre-pulsed with the peptides as described above and cultured with the harvested primed T cells. The T cells and target DCs were co-cultured for 7 hours. 1ug/ml brefeldin A (Sigma–Aldrich) was added for the final 5 hours of culture to prevent protein egress from the Golgi apparatus. Following the incubation period, the cells were labeled with the Live/Dead Fixable Stain Kit (Invitrogen, Carlsbad, CA), treated with cytofix/cytoperm and perm/wash reagents from BD Biosciences (San Jose, CA), and then labeled with Bioscience (San Diego, CA) antibodies specific for human IFNγ, TNF-α, IL-2, and granzyme B. The samples were acquired on an LSRII flow cytometer (BD Biosciences) and analyzed using FlowJo software (TreeStar, Ashland, OR). As a positive control for CD8 T cell stimulation the MHC class I control peptide pool containing a total of 32 peptides, each corresponding to a defined HLA class-I restricted T cell epitope from cytomegalovirus, Epstein-Barr virus, and influenza virus (CEF-MHC class I control peptide pool “plus” (Cellular Technology, Ltd.)) was used. CEF-MHC class I peptide pool was used at the manufacturer’s suggested concentration of 2ug/ml. Stimulation Index was calculated as the simulation seen in pre-pulsed cells stimulated peptide divided by pre-pulsed cells with no peptide added during the stimulation phase.

Supplementary Table 11. Reference and Negative Control Peptide Sequences Utilized for the In Vitro Class I HLA Allele Binding Assay.

| **Allele** | **Reference Peptide Sequence** | **Negative Control Peptide Sequence** |
| --- | --- | --- |
| **A*0101** | **IADMGHLKY** | **IPSYKKLIM** |
| **A*0201** | **GLMTTVHAI** | **IPSYKKLIM** |
| **A*0301** | **SLFRAVITK** | **IPSYKKLIM** |
| **A*2402** | **PYVSRLLGI** | **IPSYKKLIM** |
| **B*0702** | **IPSYKKLIM** | **SLFRAVITK** |
| **B*4402** | **MEVDPIGHLY** | **SLFRAVITK** |

Supplementary Table 12. Class I HLA Allele In Vitro Binding Assay Data.

For each predicted class I HLA allele epitope, the EpiMatrix Z-score and in vitro HLA binding affinity (as IC50) are shown.

| **Allele** | **Peptide Designation** | | **Sequence** | **EpiMatrix Z-score** | | **IC_50_ (nM)** |
| --- | --- | --- | --- | --- | --- | --- |
| A0101 | 311_ICS7 | | PSDKHIEQY | **2.81** | | **1997** |
| A0101 | 311_ICS2 | | PSDKHITEY | **3.56** | | **2674** |
| A0101 | 328_3D7 | | STEWSPCSV | **2.57** | | **3176** |
| A0101 | 350_CVX65 | | SADKPKDQLD | **2.25** | | **8969** |
| A0101 | 311_3D7 | | PSDKHIKEY | **3.28** | | **24837** |
| A0101 | 374_3D7 | | CSSVFNVVN | **2.11** | | **82482** |
| A0101 | 382_3D7 | | NSSIGLIMV | **2.66** | | **215471** |
| A0101 | 292_CVX337 | | NVDENANANN | **2.01** | | **666701** |
| A0101 | 357_CVX89 | | QLDYENDIEK | **2.53** | | **725826** |
| A0101 | 357_CVX154 | | ELDYENDIEK | **2.35** | | Non-Binder |
| A0101 | 357_3D7 | | ELDYANDIEK | **2.21** | | Non-Binder |
| A0101 | 109_3D7 | | NVDPNANPNV | **2.15** | | Non-Binder |
| A0101 | 370_3D7 | | KMEKCSSVF | **2.1** | | Non-Binder |
| A0101 | 336_3D7 | | VTCGNGIQV | **1.96** | | Non-Binder |
| A0101 | 316_ICS7 | | IEQYLKKIQY | **1.91** | | Non-Binder |
| A0101 | 327_3D7 (9) | | LSTEWSPCS | **1.75** | | Non-Binder |
| A0101 | 292_3D7 | | NVDENANAN | **1.65** | | Non-Binder |
| A0101 | 308_3D7 (10) | | NEEPSDKHIK | **1.64** | | Non-Binder |
| A0201 | 319_ICS2* | | YLKKIQNSL | **2.68** | | **2176** |
| A0201 | 319_ICS7 | | YLKKIQYSL | **2.95** | | **2576** |
| A0201 | 319_3D7 | | YLNKIQNSL | **2.92** | | **3047** |
| A0201 | 327_3D7 (10) | | LSTEWSPCSV | **2.29** | | **4376** |
| A0201 | 319_ICS5 | | YLKIIQNSL | **2.56** | | **4875** |
| A0201 | 315_ICS2 | | HITEYLKKI | **1.71** | | **147936** |
| A0201 | 315_3D7 | | HIKEYLNKI | **2.02** | | **259132** |
| A0201 | 376_3D7* | | SVFNVVNSSI | **2.16** | | **341608** |
| A0201 | 335_3D7* | | SVTCGNGIQV | **1.7** | | **488759** |
| A0201 | 369_3D7 (9) | | CKMEKCSSV | **1.65** | | Non-Binder |
| A0301 | 85_3D7 | | KLRKPKHKK | **3.13** | | **3056** |
| A0301 | 345_CVX79 | | RIKPGSADK | **2.55** | | **17653** |
| A0301 | 345_3D7 | | RIKPGSANK | **2.9** | | **21288** |
| A0301 | 357_3D7* | | ELDYANDIEK | **1.66** | | **556067** |
| A0301 | 357_CVX111 | | QLDYANDIEK | **2.31** | | Non-Binder |
| A0301 | 357_CVX89* | | QLDYENDIEK | **2.16** | | Non-Binder |
| A0301 | 265_3D7 | | NANPNANPNK | **2.01** | | Non-Binder |
| A0301 | 314_3D7 | | KHIKEYLNK | **1.99** | | Non-Binder |
| A0301 | 376_3D7* | | SVFNVVNSSI | **1.96** | | Non-Binder |
| A0301 | 364_3D7 | | IEKKICKMEK | **1.87** | | Non-Binder |
| A0301 | 358_3D7 | | LDYANDIEK | **1.84** | | Non-Binder |
| A0301 | 336_3D7* | | VTCGNGIQV | **1.76** | | Non-Binder |
| A2402 | 319_ICS2* | | YLKKIQNSL | **1.89** | | **11172** |
| A2402 | 377_3D7 | | VFNVVNSSI | **2.02** | | **11593** |
| A2402 | 318_3D7 | | EYLNKIQNSL | **2.63** | | **13514** |
| A2402 | 387_3D7 | | LIMVLSFLF | **2.53** | | **19280** |
| A2402 | 323_3D7* | | IQNSLSTEW | **2.19** | | **21326** |
| A2402 | 369_3D7 (10) | | CKMEKCSSVF | **2.15** | | **249581** |
| A2402 | 330_3D7 | | EWSPCSVTCG | **2.1** | | **685002** |
| B0702 | 319_ICS2* | | YLKKIQNSL | **1.95** | | **9201** |
| B0702 | 319_3D7 | | YLNKIQNSL | **1.75** | | **25553** |
| B0702 | 347_CVX36 (9) | | KPGSAGKSK | **2.13** | | **53129** |
| B0702 | 343_3D7 | | QVRIKPGSA | **1.85** | | **134253** |
| B0702 | 285_3D7* | | MPNDPNRNV | **2.9** | | **510999** |
| B0702 | 332_3D7 | | SPCSVTCGNG | **2.92** | | Non-Binder |
| B0702 | 101_3D7 | | NPDPNANPNV | **2.66** | | Non-Binder |
| B0702 | 347_3D7 | | KPGSANKPKD | **2.45** | | Non-Binder |
| B0702 | 376_3D7* | | SVFNVVNSSI | **2.1** | | Non-Binder |
| B0702 | 347_CVX69 (10) | | KPGSADKPKD | **2.07** | | Non-Binder |
| B0702 | 335_3D7* | | SVTCGNGIQV | **1.87** | | Non-Binder |
| B0702 | 131_3D7 (10) | | NPNANPNANP | **1.84** | | Non-Binder |
| B0702 | 131_3D7 (9) | | NPNANPNAN | **1.74** | | Non-Binder |
| B0702 | 370_3D7* | | KMEKCSSVF | **1.74** | | Non-Binder |
| B0702 | 107_3D7 | | NPNVDPNANP | **1.69** | | Non-Binder |
| B4402 | 360_CVX298 | | YENDIEKKI | **2.73** | | **7355** |
| B4402 | 323_3D7* | | IQNSLSTEW | **1.95** | | **136275** |
| B4402 | 371_3D7 | | MEKCSSVFN | **2** | | **142815** |
| B4402 | 316_ICS7* | | IEQYLKKIQY | **2.7** | | **156678** |
| B4402 | 356_3D7 | | DELDYANDI | **1.79** | | **276805** |
| B4402 | 294_CVX311 | | DENANANNA | **2.13** | | **895981** |
| B4402 | 329_3D7 | | TEWSPCSVT | **2.78** | | Non-Binder |
| B4402 | 308_3D7(9) | | NEEPSDKHI | **2.53** | | Non-Binder |
| B4402 | 308_CVX99 | | NEEPSDQHI | **2.46** | | Non-Binder |
| B4402 | 294_3D7 | | DENANANSA | **2.1** | | Non-Binder |
| B4402 | 285_3D7* | | MPNDPNRNV | **1.84** | | Non-Binder |
| B4402 | 308_3D7 (10)* | | NEEPSDKHIK | **1.83** | | Non-Binder |
| B4402 | 355_CVX124 | | KDELDYENDI | **1.78** | | Non-Binder |
| B4402 | 317_3D7 | | KEYLNKIQNS | **1.77** | | Non-Binder |
| B4402 | 317_ICS2 | | TEYLKKIQN | **1.65** | | Non-Binder |
| * Peptides predicted to bind two (2) HLA alleles and assessed for binding to both. | | | | | | |
|  | | | | | | |
|  | | **Class I HLA Allele IC_50_ Binding Affinity Color Key** | | |  | |
|  | | **Very High Affinity (IC_50_ < 5,000nM)** | | |  | |
|  | | **High Affinity (5,000 nM < IC_50_ < 50,000 nM)** | | |  | |
|  | | **Moderate Affinity (50,000 nM < IC_50_ < 350,000 nM)** | | |  | |
|  | | **Low Affinity (350,000 nM < IC_50_ < 1,000,000 nM)** | | |  | |
|  | | **Negligible Affinity (1,000,000 nM < IC_50_)** | | |  | |
|  | | **Non-binder (No dose-dependent inhibition)** | | |  | |

Supplementary Table 13. Peptide Binding Rates for Class I HLA Alleles.

Confirmed class I HLA allele binding for predicted epitopes ranged from 33% to 100% across the set of alleles evaluated. Overall, 58% of the predicted epitopes tested bound the class I HLA allele (in vitro) that they were predicted to bind.

|  |  |  | **Binders** | | | |  |  |
| --- | --- | --- | --- | --- | --- | --- | --- | --- |
| **Allele** | **Total Number peptides tested** | **Total Binders** | **Very High Affinity** | **High Affinity** | **Moderate Affinity** | **Low Affinity** | **Total Non- Binders*** | **Confirmed Binders (%)** |
| **A*0101** | **18** | **9** | **3** | **2** | **2** | **2** | **9** | **50** |
| **A*0201** | **10** | **9** | **5** | **0** | **3** | **1** | **1** | **90** |
| **A*0301** | **12** | **4** | **1** | **2** | **0** | **1** | **8** | **33** |
| **A*2402** | **7** | **7** | **0** | **5** | **1** | **1** | **0** | **100** |
| **B*0702** | **15** | **5** | **0** | **2** | **2** | **1** | **10** | **33** |
| **B*4402** | **15** | **6** | **0** | **1** | **4** | **1** | **9** | **40** |
| **TOTAL** | **77** | **40** | **9** | **11** | **12** | **7** | **37** | **58** |
| *Total non-binders include peptides with negligible binding and no binding. | | | | | | | | |

Supplementary Table 14. Class I PfCSP 3D7 (PFC0210c)) Epitopes Assessed by VaxDesign and the Number of Donor Responses (by HLA haplotype) with a Positive SI (SI≥2.0)

| **Peptide ID** | **Peptide Address** | **Peptide Sequence** | **CL1-#** | **Number of donor responses with SI ≥ 2** | | | | | |
| --- | --- | --- | --- | --- | --- | --- | --- | --- | --- |
|  |  |  |  | **A0101** | **A0201** | **A0301** | **A2402** | **B0702** | **B4403** |
| 85_3D7 | 85-93 | KLRKPKHKK | CL1-61 | **0** | **1** | **0** | **1** | **0** | **0** |
| 311_3D7 | 311-319 | PSDKHIKEY | CL1-62 | **5** | **1** | **2** | **2** | **2** | **2** |
| 319_3D7 | 319-327 | YLNKIQNSL | CL1-63 | **0** | **0** | **0** | **0** | **0** | **0** |

Supplementary Table 15. Functional CD8 T Cell Response Profiles for Class I PfCSP 3D7 Epitopes Assessed by VaxDesign

| **Marker** | **IFN-γ** | | | | | | **TNF-α** | | | | | | **IL-2** | | | | | | **Granzyme** | | | | | |
| --- | --- | --- | --- | --- | --- | --- | --- | --- | --- | --- | --- | --- | --- | --- | --- | --- | --- | --- | --- | --- | --- | --- | --- | --- |
| **HLA** | **A1** | **A2** | **A3** | **A24** | **B7** | **B44** | **A1** | **A2** | **A3** | **A24** | **B7** | **B44** | **A1** | **A2** | **A3** | **A24** | **B7** | **B44** | **A1** | **A2** | **A3** | **A24** | **B7** | **B44** |
| **CL1-61** |  |  |  |  |  |  |  |  |  |  |  |  |  |  | **1** |  |  |  |  |  |  |  |  |  |
| **CL1-62** | **4** |  | **1** | **1** | **1** | **1** | **3** |  |  | **1** |  | **1** | **4** |  | **1** | **1** | **1** | **1** | **1** |  |  |  |  |  |
| **CL1-63** |  |  |  |  |  |  |  |  |  |  |  |  |  |  |  | **1** |  |  |  |  |  |  |  |  |
